# Valorization of Residual Babassu Mesocarp To Obtain Lipases and Laccases by Solid-State Fermentation

**DOI:** 10.1021/acsomega.5c03496

**Published:** 2025-06-30

**Authors:** Tamires N. dos Anjos, Selma G. F. Leite, Ivana C. R. Leal, Ivaldo Itabaiana

**Affiliations:** † Department of Biochemical Engineering, School of Chemistry, Federal University of Rio de Janeiro, Rio de Janeiro 21941-909, Brazil; ‡ Department of Natural Products and Food, Faculty of Pharmacy, Federal University of Rio de Janeiro, Rio de Janeiro 21941-902, Brazil

## Abstract

The growing life expectancy of the world’s population has demanded more significant inputs of food and energy, stimulating the industrialization of agriculture, with the subsequent generation of residual lignocellulosic biomass of various kinds, where new technologies for its valorization and reinsertion into the production chain are essential. Babassu (*Attalea speciosa* Mart. Ex Spreng), a palm native to the north and northeast of Brazil, plays a critical role in the economic development of these regions, with notable applications in the oil industry, generating large quantities of residual babassu mesocarp (BM). In this study, BM was investigated as a matrix for the production of lipases and laccases, enzymes of great biotechnological importance, by solid-state fermentation (SSF) with *Trichoderma harzianum* (IOC4042) and *Geotrichum candidum* (CCT1205). The in natura (IN-BM) and defatted (DEF-BM) materials were fermented with hydration solutions containing 3–7 salts, combined with glucose or sucrose. The best enzyme activity results were obtained with solutions of 7 salts and glucose, where maximum lipase activity was demonstrated by *G. candidum* IN-BM (33.14 ± 0.017 U·mL^–1^) and the laccase activity by *T. harzianum* in DEF-BM (0.524 ± 0.016 U·mL^–1^). Laccase production was also demonstrated for the first time with *G. candidum* in DEF-BM (0.441 ± 0.069 U·mL^–1^). The results showed for the first time that residual babassu mesocarp is a promising matrix for the bioproduction of lipases and laccases by *G. candidum* and *T. harzianum* through a simple and competitive methodology, adding value to an agro-industrial waste.

## Graphical abstract

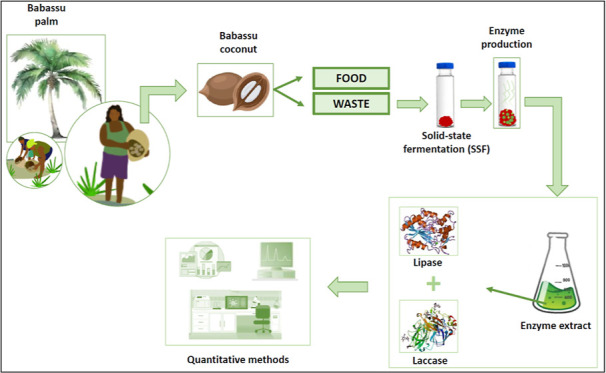

## Introduction

Agriculture has become a strategic sector due to the growing global demand for food and energy, intensifying the industrialization and processing of various agricultural products.^1^ This has led to profound environmental impact and large quantities of agro-industrial waste being disposed of inappropriately, requiring new technologies for reuse and obtaining compounds with greater added value, reinserting them into the production chain.^2^ This practice reduces greenhouse gas (GHG) emissions, pollution and the proliferation of vectors, contributing to sustainable development, in line with the principles of biorefineries and the SDGs (Sustainable Development Goals).^1,3^ According to United Nations Environment Program (UNEP), around 1.05 million tons of agricultural commodities with food potential were discarded in 2022, and in Brazil, about 5% by weight of BM was reused.^4^ Most of this waste is lignocellulosic biomass—made up of cellulose, hemicellulose, lignin and other organic components—which can be assimilated by various microorganisms to produce bioactive compounds with applications in the chemical, food and pharmaceutical sectors, among others.^5^

One of the most generated agro-industrial residues in Brazil is babassu mesocarp (BM), which comes from the babassu palm (*Attalea speciosa* Mart. Ex Spreng), a plant native to the North, Northeast, and Center-West regions, whose oil extraction from its kernels has a substantial economic influence. BM is rich in starch, cellulose, proteins, lipids, and mineral salts.^6^ The babassu coconut, from which BM is extracted, is structurally composed of four parts: fibrous epicarp (11–13%), mesocarp (20–23%), woody endocarp (57–63%), and kernel (7–9%).^7^ Usually, the residual BM is obtained after separating the kernel for oil extraction and can undergo other processing, mainly destined for meal production for animal feed.^6^ In 2022, babassu production reached 30,478 tons in Brazil, where about 5% by weight of MB was generated, demanding new alternative forms of reuse.^8^ Given its nutritional and biochemical richness, BM has the potential to be exploited through fermentation for the biotechnological production of high-value-added compounds. Our group has previously studied this biomass’s potential for producing aroma compounds,^7^ setting a precedent for future studies. In some processes, the presence of oils or fats in the biomass can hinder the fermentation process by inhibiting the microorganism’s metabolism and access to the desired fractions. In this way, pretreatment to defatted the biomass can be an auxiliary method for fermentation.^7^

In biotechnological processes, various microbial transformations are carried out through the production and secretion of enzymes. These bioproducts are of great importance in the industrial scenario, as they operate under milder reaction conditions, are selective in obtaining products and contribute to the generation of more environmentally favorable conditions, which makes the enzyme market is an expanding field.^9^ Among the enzymes of great industrial value are lipases (EC 3.1.1.3) and laccases (EC 1.10.3.2), biocatalysts recognized for their versatility and the range of chemical reactions they catalyze.^5,10^

Lipases are hydrolases with broad biotechnological and industrial applicability whose natural reaction is the hydrolysis of triacylglycerols, with the end product being the generation of the corresponding fatty acids and glycerol.^10^ In water-restricted environments, lipases can catalyze the reverse reaction. In addition to hydrolysis and esterification, lipases catalyze transesterification, amidation, kinetic resolution, and other reactions, with chemo-, regio- and enantioselectivity.^11^ In addition, they are active in organic solvents, do not require cofactors, and can be produced relatively quickly. This makes them extremely attractive, being the third largest group of enzymes based on market value.^12^ According to Guerrand (2017),^13^ around 75% of industrialized enzymes are hydrolases, and 90% are of microbial origin derived from fermentative processes. Microbial lipases have great catalytic potential, and the main advantage of their use over the conventional route is the possibility of using oily raw materials with a high acidity index for their production.^14^

Laccases are multicopper oxidoreductases that use the redox potential of their active site to catalyze the oxidation of aromatic and nonaromatic compounds, reducing the oxygen molecule to water.^15^ These enzymes, present for example in wood-degrading fungi, can oxidize a wide variety of organic and inorganic compounds and, due to this characteristic, have a wide range of biotechnological and industrial applications, such as the degradation of xenobiotics, pharmaceutical residues, textile dye effluents and even in food processing and paper production.^16^ Fungal laccases are part of the enzymatic system capable of degrading lignin in agro-industrial residues, making cellulose and hemicelluloses more accessible for various bioprocesses.^15,17^ Thus, the growing interest in obtaining these enzymes, combined with the high cost of commercial preparations, makes biomass valorization a robust strategy for obtaining a competitive biocatalyst at a lower price.

According to Markets and Markets,^18^ the global enzyme market was estimated at 14 billion dollars in 2024 with projections to reach 20.4 billion by 2029, with a compound annual growth rate (CAGR) of 7.8%. The global lipase market was estimated at 612.6 million in 2023, and is expected to reach 1631.2 million by.^19^ The global laccase market was 3 million in 2023 and is expected to reach 4.37 million by 2032.^20^ In this trend, obtaining new enzymes from microbial sources could represent a biotechnological breakthrough, allowing the discovery of innovative properties and kinetic characteristics with potential for application and industrial competitiveness. This process makes the most of agro-industrial waste as a fermentation substrate and can result in simpler and more economical protocols.^21^ Solid-state fermentation (SSF) has thus been highlighted as a viable, low-cost technology for enzyme production, using solid waste as BM, which offers favorable physical and chemical attributes for microbial growth and simulates the natural habitat of these microorganisms.^9,10^

Among the microorganisms of great importance in industrial biotechnology are the filamentous fungi *Trichoderma harzianum* and *Geotrichum candidum*, which our research group has studied to develop bioprocesses for the valorization of biomass and to obtain various value-added compounds.^7,22,23^ To date, there are no reports in the literature involving the application of *T. harzianum* and *G. candidum* in the valorization of BM, where we reported for the first time the ability of these microorganisms to produce lipases and lacases. In addition, *T. harzianum* and *G. candidum* are GRAS (Generally Recognized as Safe), and their use in the production of enzymes allows these molecules to be used in various industrial sectors.^22^ Therefore, as a continuation of our efforts, this study aimed to investigate the production of lipases and laccases from the valorization of residual BM by SSF, using *T. harzianum* IOC 4042 and *G. candidum* CCT1205 as agents.

## Experimental Section

### Babassu Mesocarp Obtaining and Processing

The raw BM was obtained in the rural area of Ariquemes, Rondônia—RO, Brazil (−9.916842, −62.971829), specifically from the babassu palm (Figure 1A), which produces varying quantities of babassu bunches (Figure 1B). The babassu coconut (Figure 1C) was collected, washed in distilled water, and broken for manual extraction of the mesocarp (Figure 1D).

**Figure 1:**
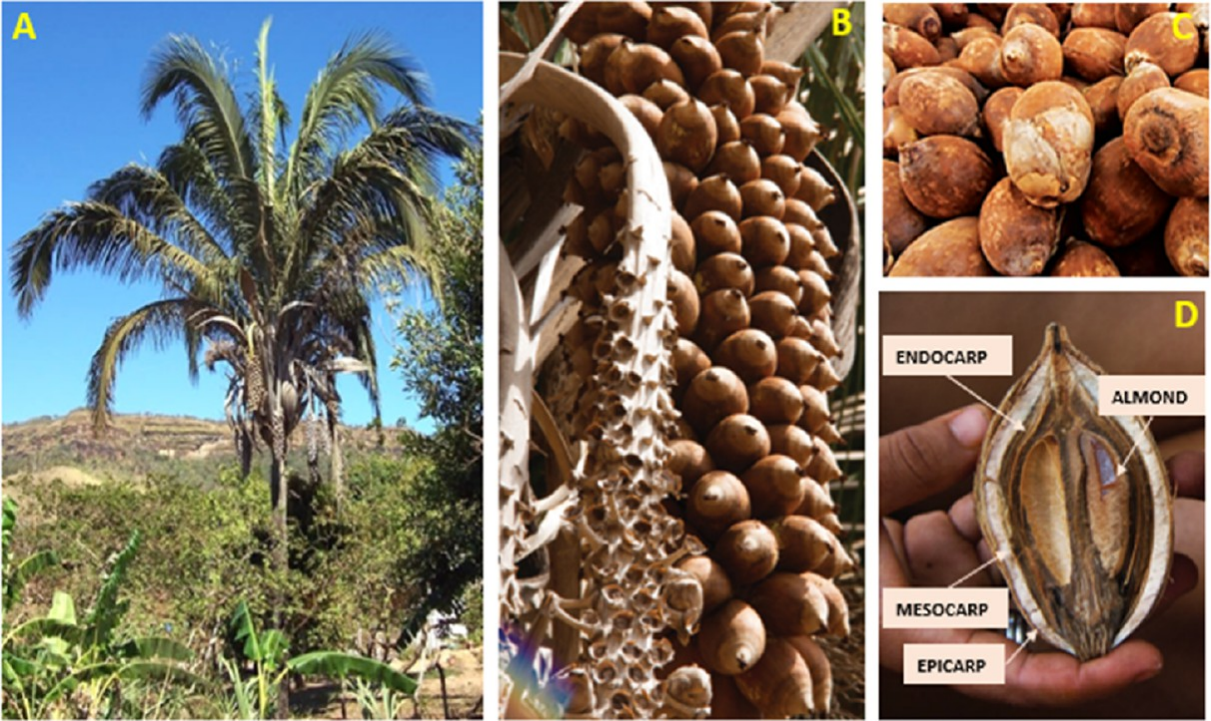
Obtaining the raw babassu mesocarp: (A) babassu palm; (B) babassu coconut bunch; (C) babassu coconut; (D) sectioned babassu coconut, in which its structural composition can be seen: epicarp, mesocarp, endocarp and almonds. Adapted with permission from [Muniz, C. P. L.; Santos, A. M. (Org.) (2016) Universo Cultural Da Palmeira De BabaçU. 1st ed. Brasi’lia: IPHAN, V. 1. 150P.] Copyright [2016/Cejane Pacini Leal Muniz]­[Instituto Do Patrimônio Histo’rico E Arti’stico Nacional (IPHAN)/Instituto Do Patrimônio Histo’rico E Arti’stico Nacional (IPHAN)].

The material was dried for 5 days in an oven at 40 °C and then processed in a Wiley knife mill (TE-680) to a particle size of 10 Tyler mesh. The material was then sieved to standardize the particle size, selecting the fractions not retained on the 14 mesh sieve (particles with diameters smaller than 1.19 mm).

### Babassu Mesocarp Pretreatments

Initially, part of the sieved raw babassu mesocarp (Figure 2A) was subjected to a Soxhlet degreasing process to investigate the influence of the lipid fraction on fermentation. To do this, 500 g of the powder was thoroughly washed with distilled water (5 times 200 mL), dried in an oven at 25 °C for 24 h, and then treated with 95% hexane as a solvent in a Solab Soxhlet apparatus (100 g of support/200 mL of solvent). The temperature in the extractor was kept constant during a reflux period of 4 h, within the boiling range of hexane (63.7 °C–69 °C). The organic phase containing the extractives was concentrated under vacuum, and the defatted solid was dried at room temperature in an exhaust hood for 24 h and called defatted babassu mesocarp (DEF-BM) (Figure 2C). The solid that did not undergo this process was initially called *in natura* babassu mesocarp (IN-BM) (Figure 2B). Both IN-BM and DEF-BM were applied in the subsequent fermentation steps, aiming for comparative data.

**Figure 2:**
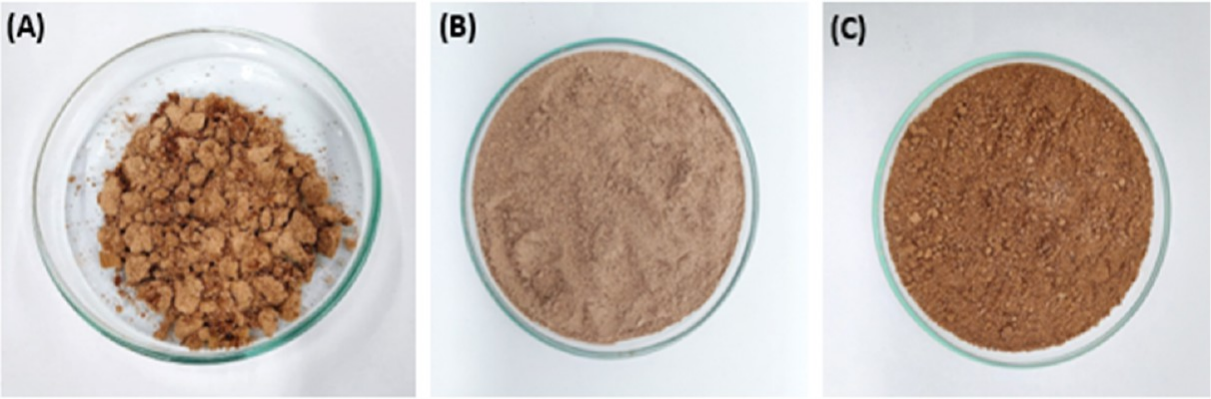
Physical aspects of BM. (A) Raw babassu mesocarp after extraction from the fruit; (B) IN-BM; and (C) DEF-BM.

### Bromatological and Physical–Chemical Analysis

#### Bromatological Analysis

The quantification of starch, proteins, hemicellulose, cellulose and lignin in the BM was carried out by the Bromatological Analysis Laboratory (LABBROM) of the State University of Southwest Bahia (UESB) and the Food Analysis Laboratory (LANA) of the Federal University of Maranhão, according to the analytical standards of the Adolfo Lutz Institute (IAL, 2008),^24^ the AOAC analytical methods (AOAC INTERNATIONAL, 1995)^25^ and the neutral detergent fiber method proposed by VAN SOEST (1994).^26^ All the analyses were carried out in triplicate.

#### Moisture Content, pH, and Ash

The moisture content of the waste was determined using 200 mg of each sample at 160 °C in a closed chamber, using a model MX-50 balance. For the calculation, 10 repetitions of the analysis were carried out. The pH was determined according to the official method (AOAC INTERNATIONAL, 1997).^27^ For this, 500 mg of IN-BM and DEF-BM before and after the best fermentation conditions were soaked in 5 mL of distilled water and homogenized in a vortex model 45–2810, Kasvi Basic, for 30 s. The pH was measured in the supernatant of the samples 3 min after homogenization. The analysis was carried out in triplicate. The ash content was determined by directly incinerating the waste in a muffle furnace at 550 °C until constant weight (IAL, 2008).^24^

#### Determination of Total Reducing Sugars

Total reducing sugars (TRSs) were analyzed in IN-BM and DEF-BM (before fermentation). 2.0 g of each solid was subjected to constant orbital agitation in 100 mL of distilled water for 120 h at 150 × g in a refrigerated Shaker incubator model NT715, Novatecnica, at 25 °C. Aliquots of 2 mL of each system were taken every 24 h for 7 days. All samples were centrifuged at 13,000*g* for 5 min in a Sorval legend micro 17 microcentrifuge (Thermo Scientific). The supernatant was collected for the dosage of ARTs using the DNS (dinitro salicylic acid) reagent methodology established by Miller (1959):^28^ 100 μL of each sample were incubated with 300 μL of the DNS reagent for 5 min in a boiling bath at 100 °C. Then 1.0 mL of distilled water was added and the absorbance of the samples was measured at 540 nm using a Biospectro model SP-220 spectrophotometer. All analyses were carried out in triplicate.

### Microorganisms and Culture Propagation

The fungal strains *T. harzianum* IOC 4042 and *G. candidum* CCT1205 were kindly provided by the Mycology Department of the Oswaldo Cruz Institute (FIOCRUZ). The cultures were maintained in test tubes containing PDA (potato dextrose agar) medium, according to Martins (2003).^29^ The samples were then replanted and incubated in a greenhouse for 7 days at 30 °C and refrigerated at 4 °C. *T. harzianum* was propagated by spreading its spores in Petri dishes containing (g·L^–1^): malt extract, 10; glucose, 4.0; agar–agar, 13.0; yeast extract, 0.75 and peptone, 0.75 at pH 5.0. For *G. candidum*, propagation occurred in Petri dishes containing (g·L^–1^): glucose, 5.0; agar, 13; yeast extract, 0.50; peptone, 0.50; and pH 5.0. Both propagations were incubated for 7 days at 30 °C.

### Solid State Fermentation

The investigation into the production of lipases and laccases by *T. harzianum* and *G. candidum* strains by SSF was carried out using IN-BM and DEF-BM as substrates, in which the initial inoculum was prepared from a suspension of fungal spores in saline solution (NaCl 0.9% p·v^–1^), which was homogenized in a vortex and used to count the number of spores in a Neubauer chamber (Kasvi), using a Primo Star optical microscope model 4155 (Zeiss). The fermentation process was done in sterile 20 mL Erlenmeyer flasks containing 2.0 g of each dried residue. Each material was autoclaved at 1 atm for 20 min, soaked in 2.0 mL of the appropriate hydration solution, previously autoclaved, and 1.0 mL of spore suspension (10^5^ spores·mL^–1^). For both IN-BM and DEF-BM, the hydration solutions investigated were: (1) distilled water (DW); (2) nutrient solution containing three salts (NS3S); (3) nutrient solution containing seven salts (NS7S), (4) nutrient solution with glucose (NSG) and (5) nutrient solution with sucrose (NSS). The NS3S and NS7S nutrient solutions had the following compositions, according to Ramos et al. (2008)^30^ (mg·g^–1^ of residue): NS3S—(NH_4_)_2_SO_4_, 0.94; KH_2_PO_4_, 1.0; MgSO_4_·7H_2_O, 5.0; NS7S—(NH_4_)_2_SO_4_, 0.94; yeast extract, 1.0; MgSO_4_·7H_2_O, 5; KH_2_PO_4_, 1.0; KCl, 0.5; CaCl_2_·2H_2_O, 0.008; FeSO_4_·7H_2_O, 0.01; ZnSO_4_·7H_2_O, 0.001. The combination of NS3S and NS7S was also compared with NSG and NSS (30 mg·g^–1^ of residue). The flasks were then sealed with a cotton cloth and kept in a Visome Plus—VCC300 climate chamber for 7 days at 30 °C and 90% humidity. The fermentations were carried out in triplicate with a bed height of 1.5 cm. Samples were taken every 24 h, the moisture and pH of the fermented medium were evaluated, and enzymes were extracted for later determination of activity.

### Determination of Moisture and pH

The moisture content of the solid supports was determined on a Shimadzu MOC63 V moisture balance, where 1 g of each residue was analyzed before and after the fermentation process at 105 °C constantly until there was no change in weight (IAL, 2008).^24^ The pH of the samples was analyzed with the supernatant in a bench potentiometer.

### Obtaining the Enzyme Extracts

The enzyme extracts were obtained by extracting the IN-BM and DEF-BM biomass after fermentation with sodium citrate buffer 50 (pH 5.0) in a ratio of 1:5 (m·v^–1^), at 200 × g and 30 °C for 20 min in Erlenmeyer flasks. The mixture was then pressed, and the crude extract obtained was centrifuged at 3400*g* for 5 min. The supernatant was collected and stored under refrigeration for analysis of enzymatic activity.

### Enzymatic Assays

#### Determination of Lipolytic Activity

The *T. harzianum* and *G. candidum* strains were first investigated using the qualitative Rhodamine B assay for their lipolytic potential. To do this, the strains were activated for 7 days in the propagation medium described above. Plates were then prepared with 20 mL of solid rhodamine medium (Kouker and Jaeger (1987) modified^31^) containing (g·L^–1^): yeast extract, 0.5; nutrient broth, 10; NaCl, 4.0; agar, 10; in addition to olive oil 2.5% (p·v^–1^); rhodamine B 0.001% (m·v^–1^); and distilled water to complete the volume. The medium was autoclaved at 1 ATM for 15 min, followed by adding rhodamine B. The fungi were inoculated individually in the center of the gelled plates with a microbiological loop. The plates were incubated for 7 days in an oven at 25 °C and, every 24 h, the formation of a fluorescent halo around the inoculation point visible under ultraviolet light at 365 nm was investigated as a product of the hydrolysis of olive oil triacylglycerols. The lipase activity in the fermented products was investigated comparatively using spectrophotometric and titration methods.

#### Spectrophotometric Hydrolytic Method

The lipolytic activity of the IN-BM and DEF-BM enzyme extracts obtained by the two strains was investigated using the *p*-nitrophenyl laurate hydrolysis method in DMSO/acetonitrile 1:1 (2.5 mM). To determine lipolytic activity, 25 μL of each enzyme extract was added to a cryotube, 1.1 mL of sodium phosphate buffer (25 mM, pH 7.0), and then 125 μL of the substrate, which was kept in a thermostatic bath at 30 °C for 5 min. The reaction was monitored in a spectrophotometer Bel Photonics VM5, checking the absorbance at 412 nm every 30 s for 5 min. Control tests were carried out without enzymes. Enzymatic activity was determined by the concentration of ρ-nitrophenol in mol·L^–1^, based on the molar absorptivity coefficient of ρ-nitrophenol, obtained by drawing up a calibration curve at 410 nm. A unit of enzyme activity (U) was defined as the amount of enzyme needed to produce one μmol of ρ-nitrophenol per minute, according to eq 1.

1 $$A_{Lip}\left(U\;{mL}^{-1}\right)=\frac{Abs\times D\times f\times 1000}{t\times V}$$

where: *A*_LIP_ = lipase activity (U mL^–1^), Abs = absorbance in the time interval elapsed during the phase of linear increase in absorbance, *D* = dilution of the enzyme solution, *f* = conversion factor (0.1062 μmol mL^–1^), *V* = volume of enzyme solution used in the test (mL), *t* = reaction time (min).

#### Titrimetric Lipolytic Method

For lipolytic activity using the titrimetric method, an emulsion of olive oil (5% m·v^–1^) and arabic gum (5% m·v^–1^) in sodium phosphate buffer (100 mM and pH 7.0) was prepared by stirring in a mixer for 3 min. After this, 1 mL of the appropriate enzyme extract was added to 19 mL of the emulsion and incubated at 35 °C and pH 7.0 for 15 min in a water bath at 200 × g. The reaction was stopped by adding a solution of acetone-ethanol (1:1 v·v^–1^). The released fatty acids were titrated with a 0.04 M NaOH solution in a previously calibrated Mettler Toledo Compact G20S automatic titrator until a pH of 11.0 was reached. The reaction blanks were carried out without an enzyme. Under the test conditions, one unit of enzyme activity (U) was equivalent to 1 μmol of fatty acid per minute. Thus, the enzyme activity was calculated using eq 2.

2 $$A_{Lip}\left(U\;{mL}^{-1}\right)=\frac{\left(V_{a}-V_{b}\right)\times M\times 1000}{t\times V}$$

where: *A*_LIP_ = lipase activity (U mL^–1^), *V*_a_ = volume of NaOH solution used to titrate the sample (mL), *V*_b_ = volume of NaOH solution used to titrate the blank (mL), *M* = molarity of the NaOH solution (mol L^–1^), *t* = reaction time (min), *V* = volume of sample (mL).

#### Laccase Activity

The quantification of laccase activity in the enzyme extracts of IN-BM and DEF-BM was investigated by comparing the oxidation methodologies of ABTS (2,2′-azino-bis-3-ethylbenzothiazoline-6-sulfonic acid) and guaiacol as substrates. The first test was carried out according to Magalhães et al. (2018):^32^ the reaction was conducted in test tubes with the addition of 1.7 mL of 100 mM sodium acetate buffer (pH 5.5), 100 μL of each enzyme extract and 200 μL of 10 mM ABTS aqueous solution. The system was kept in a water bath at 30 °C for 5 min, followed by measurement of the absorbance at 420 nm. One unit of enzymatic activity (U) was defined by the enzymatic oxidation of 1 μmol of substrate per minute, and the laccase activity (U mL^–1^) was calculated according to eq 3.

3 $$A_{Lac}\;\left(U\;{mL}^{-1}\right)=\frac{V_{f}\times Abs\times D}{\varepsilon \left({mM}^{-1}\;{cm}^{-1}\right)\times CO\times V_{a}\times t}$$

where: *A*_Lac_ = laccase activity (U mL^–1^), *V*_f_ = reaction volume (mL), Abs = absorbance in the time interval during the phase of linear increase in absorbance, *D* = dilution factor, ε = molar extinction coefficient of ABTS at 420 nm (36,000 mM^–1^ cm^–1^), CO = optical path (cm), *V*_a_ = sample volume (mL), *t* = reaction time (min).

The guaiacol oxidation assay followed a methodology adapted from Kalra et al. (2013):^33^ in cryotubes, 100 μL of each enzyme extract, 100 μL of 10 mM aqueous guaiacol solution, and 800 μL of 10 mM sodium acetate buffer (pH 4.5) were added, and this system was incubated in a water bath at 30 °C for 15 min, followed by an absorbance reading at 450 nm. One unit of laccase activity (U) was defined as the amount of enzyme required to oxidize 1 μmol of guaiacol per minute, and the activity (U mL^–1^) was calculated using eq 4.

4 $$A_{Lac}\left(U\;{mL}^{-1}\right)=\frac{Abs\times V}{\varepsilon \left({mM}^{-1}\;{cm}^{-1}\right)\times V_{a}\times t}$$

where: *A*_Lac_ = laccase activity (U mL^–1^), Abs = absorbance, *V* = final reaction volume (mL), *V*_a_ = sample volume (mL), *t* = incubation time (min), ε = extinction coefficient of guaiacol (48,000 mM^–1^ cm^–1^).

#### Statistical Analysis

The differences between the lipase and laccase activity experiment replicates were subjected to statistical analysis. The results were expressed as mean, median, variance, and standard deviation for each group of samples. The samples were considered independent, and a significance level of 5% (*p* < 0.05), degree of freedom 2, and 95% confidence interval were used. The data was tabulated using Microsoft Excel version 2019.

#### Protein Quantification and Enzyme Profiles

The protein concentration in the enzyme extracts during the fermentations was determined according to an adaptation of the Bradford method (1976)^34^ with an absorbance reading at 595 nm, based on a bovine serum albumin (BSA) standard curve (Figure S1 in the Supporting Information). The enzymatic profile of the extracts obtained under the best fermentation conditions for the expression of lipases and laccases was evaluated by 12% (p·v^–1^) polyacrylamide gel electrophoresis under denaturing conditions with sodium dodecyl sulfate (SDS-PAGE), according to the methodology described by Laemmli (1970):^35^ A 15 μg aliquot of the crude extracts was precipitated with trichloroacetic acid (TCA). The samples were heated for 5 min at 95 °C, then incubated at room temperature for 90 min at 150 V, applying a current of 20 mA, using Mini-PROTEAN Tetra Cell equipment (Bio-Rad). The running buffer was Tris–HCl 50 mM, pH 6.8; glycine 150 mM, SDS 0.1% (w·v^–1^) and the molecular weight standard Bio-Rad Kaleidoscope Standards. Once the run was complete, the gels were added to 250 mL of a fixing solution of 125 mL methanol, 25 mL acetic acid and 100 mL distilled water and refrigerated for 24 h. After this stage, the gels were stained in a solution consisting of 40% (v·v^–1^) methanol, 10% (v·v^–1^) glacial acetic acid, 0.1% (m·v^–1^) Coomassie Blue (CBB R-250) and distilled water, for 3 h under orbital agitation at 20 × g. The gels were washed with distilled water, decontaminated in 50 mL of methanol and 35 mL of acetic acid and fixed again with a fixative solution for 24 h.

#### Analysis of the Surface of the Fermented Supports—Scanning Electron Microscopy and Analytical Magnifying Glass

Scanning electron microscopy (SEM) and analysis with an analytical magnifying glass were carried out to verify the adherence of the microorganisms to the matrices studied under the best fermentation conditions. To investigate the hydrolysis of granular starch, samples of IN-BM in natura and DEF-BM were analyzed as a negative control. SEM was conducted using a Hitachi (TM—3030 Plus) scanning electron microscope with coupled EDS (Bruker). Before analysis, the samples were subjected to a gradual drying pretreatment and metallization with gold (99.99%) in an Emitech metallizer (K550). The analytical loupe used was the Zeiss SteREO Discovery V8 loupe. Both analyses were conducted at the Multiuser Laboratory for Technological Characterization (LMCT) of the Centre for Mineral Technology (CETEM) at Federal University of Rio de Janeiro (UFRJ).

## Results and Discussion

### Characterization of Agro-Industrial Waste Supports

This work began with the physicochemical characterization of the IN-BM and DEF-BM supports (Table 1) to understand the nutrient contents important for developing the bioprocess and ascertain the efficiency of the degreasing process to obtain DEF-BM. Moisture data was represented on a humid basis, and the other components were represented on a dry basis.

**Table 1:** Physico-chemical Characterization of IN-BM and DEF-BM

	mass content (%)	
component	IN-BM	DEF-BM
proteins	9.99	6.62
lipids	16.96	5.02
ash	2.26	1.57
total carbohydrates (TC)	70.79	78.79
fibrous carbohydrates (FC)	42.42	51.42
non-fibrous carbohydrates (NFC)	28.37	27.37
hemicellulose	13.25	13.03
cellulose	17.52	9.96
lignin	20.09	28.43
starch	83.70	75.09
humidity^a^	12.87	9.28

a Expressed on a humid basis.

As observed, IN-BM and DEF-BM had a majority starch composition (83.70%) and (75.09%), respectively. The high content of this carbohydrate can be an efficient carbon source for various fermentative processes, as microorganisms can produce and secrete amylases to degrade this substrate into oligomers and glucose. Microorganisms that can degrade the lipid fraction and lignin can also be used to pretreat the biomass to obtain a starch-rich fraction. Investigating this hypothesis was also the focus of this study since the two fungal strains explored did not show amylase activity in a preliminary screening (results not shown). The starch content obtained for IN-BM was slightly higher than the values found in the literature but was similar to that found in the study by Oliveira (2018)^36^ (80.66%), while in DEF-BM, the starch content was around 7% lower. These results were also around 39% (IN-BM) and 25% (DEF-BM) higher than those observed in the study by Cinelli (2012)^37^ for babassu flour (60.05%) and 25% (IN-BM) and 12% (DEF-BM) higher than those observed in the study by Silva (2011)^38^ for BM (66.92%). The starch content obtained in the research by Maniglia (2017)^39^ (84.57%) was very similar to the content found in IN-BM in this study.

A representative amount of crude protein (9.99%) was also observed in IN-BM and (6.62%) in DEF-BM, higher than that observed in the study by Couri and Giada (2016)^40^ (3.40%). The values of TC, CF, and NFC in the supports were (70.79%, 42.42%, and 28.37%, respectively) in IN-BM and (78.79%, 51.42%, and 27.37%, respectively) in DEF-BM. Carbohydrate values equivalent to around 52% for this residue have been reported in the literature.^41^ The characterization of 2 babassu mesocarp flours carried out in the study by Sousa et al. (2014)^42^ obtained for BMFI (babassu mesocarp flour I) and BMFII (babassu mesocarp flour II), respectively, FC content equivalent to (60.20% and 44.28%) and NFC content equivalent to (44.02% and 32.75%). The CF content of FMBI was higher than that obtained from IN-BM and DEF-BM, and the CF content of BMFII was higher only when compared to IN-BM. The NFC content of BMFI and BMFII was higher than that of IN-BM and DEF-BM. These results characterize IN-BM and DEF-BM as highly fibrous waste. The ash content of IN-BM (2.26%) was double that found in the study by Maniglia and Ta’pia-Blacido (2016)^43^ (1.13 ± 0.12 g/100 g of sample) and, compared to the same survey, the ash content of DEF-BM (1.57%) was slightly higher.

When characterizing the babassu mesocarp, Silva (2011)^38^ obtained protein (7.36%), carbohydrate (72.20%), ash (0.780%), and moisture (12.02%) contents. These values were slightly closer to those obtained in this study. As for the moisture content, the study mentioned above, and the studies by Oliveira (2018)^36^ (13.34%) and Couri and Giada (2016)^40^ (13.07%) showed results very similar to those observed in IN-BM (12.87%) and results higher than those observed in DEF-BM (9.28%). This result is expected, as the Soxhlet degreasing process was carried out under heat and with ethanol, which can form an azeotropic mixture with water and provide part of its extraction from the matrix. The lipid content observed in IN-BM (16.96%) and DEF-BM (5.02%) was much higher than that reported by Couri and Giada (2016)^57^ (0.27%) and Silva (2011) (0.08%).^38^ However, the methodology used in this study efficiently removed more than 70% of the fat content in DEF-BM. Thus, DEF-BM was evaluated and compared with IN-BM as matrices for the production of lipases and laccases. Sousa et al. (2014)^42^ quantified the hemicellulose (16.17%), cellulose (26.07%), and lignin (21.16%) contents for babassu mesocarp flour, which are in the same range as those found by our group. The expected variation in the raw material components is related to the factors of cultivation, extraction, maturation, soil type, and processing.^36,39,41^

The results obtained in this study show that defatting the biomass resulted in a reduction in the contents of starch (10%), cellulose (44%), ash (30%), lipids (70.4%), proteins (32%), hemicellulose (2%) and NFC (3%) and an increase in the contents of TC (11%), lignin (41%) and FC (21%), making them potential differential inductors for lipases and lacases.

The supports were also characterized in terms of the content of TRSs and glucose present in this raw material, which was equivalent to (0.525 g·L^–1^ and 0.317 g·L^–1^), respectively, in IN-BM and (0.623 g·L^–1^ and 0.402 g·L^–1^), respectively, in DEF-BM at the end of 7 days (168 h) of analysis (in the absence of microorganisms). Due to this concentration of readily available sugars and the starch content, enzyme production was investigated in subsequent studies with and without supplementation of carbohydrates and salts. The pH analyses showed that IN-BM and DEF-BM had values equivalent to 4.7 and 4.1, respectively, values compatible with the growth of the fungal species studied.

### Evaluation and Quantification of Lipase Activity

The species *T. harzianum* IOC 4042 and *G. candidum* CCT1205 were investigated for their lipolytic potential using the qualitative rhodamine B assay, in which the lipase secreted by the microorganisms hydrolyzes the triglyceride present in olive oil and the free fatty acids released from a colorimetric complex with the dye. Growth was observed in the medium containing rhodamine B only after 48 h of cultivation for both microorganisms (Figure 3). However, under ultraviolet light, no fluorescent halo was observed during this period, only at 72 h (Figure 3A,B), where a fluorescent halo was observed around the central dot (Figure 3A1) and fluorescent dots along the length of the plate (Figure 3B1), suggesting lipolytic activity. At 96 h, there was a slight increase in fluorescence on the plates of both fungi (Figure 3A2,B2), which also happened at 120 h (Figure 3A3,B3), remaining constant until 192 h. The fact that these strains showed cell growth in this restricted medium containing only triacylglycerols as a carbon source is a qualitative indication of their ability to secrete lipases and subsequently hydrolyze and release fatty acids for the rhodamine B reaction. In this way, both strains were characterized as lipolytic, and the quantification of the lipase activity of each microorganism was determined in the following steps. Canseco-Pérez et al. (2018)^44^ also obtained favorable results for lipase activity in *T. harzianum* using rhodamine B 0.001% (w·v^–1^). However, for *G. candidum*, this activity is being reported for the first time.

**Figure 3:**
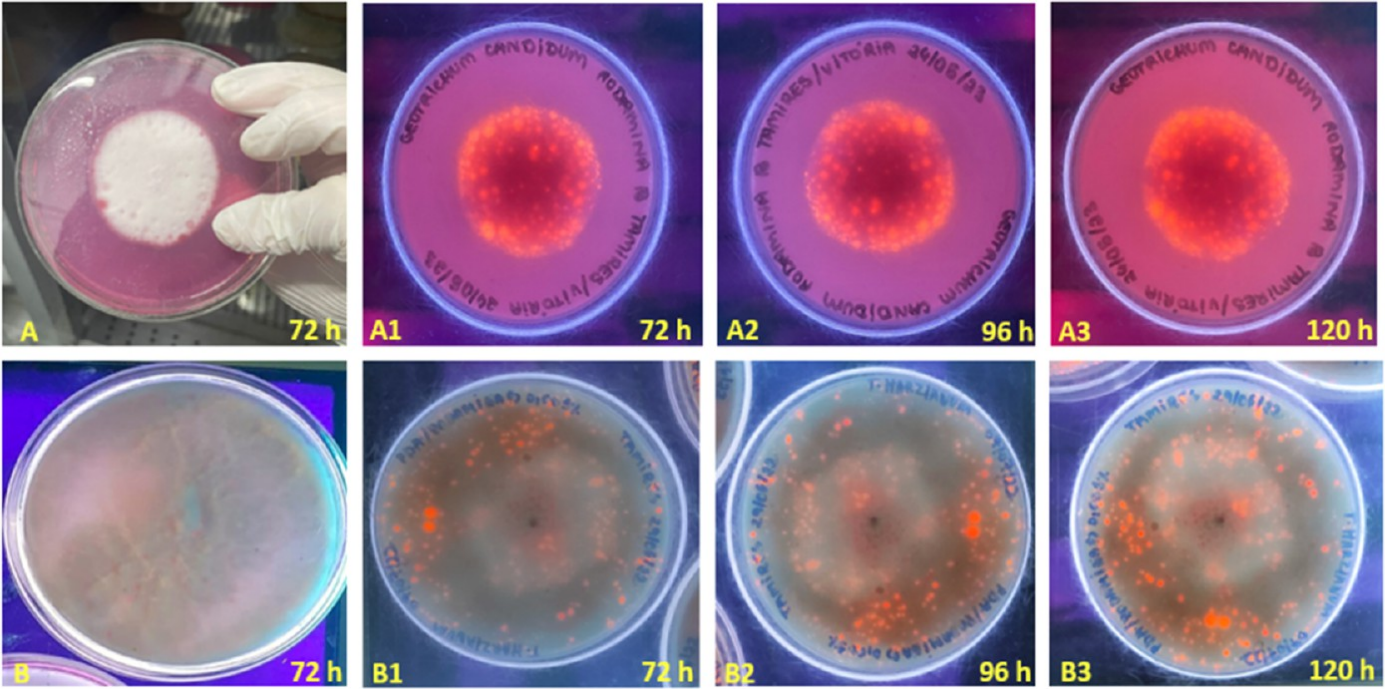
Qualitative assay of lipase activity of *T. harzianum* and *G. candidum* in a medium containing rhodamine B dye. (A) Lipase activity of *G. candidum* after 72 h; (A1) lipase activity of *G. candidum* after 72 h; (A2) lipase activity of *G. candidum* after 96 h; (A3) lipase activity of *G. candidum* after 120 h; (B) lipase activity of *T. harzianum* after 72 h of cultivation; (B1) lipase activity of *T. harzianum* after 72 h; (B2) lipase activity of *T. harzianum* after 96 h; (B3) lipase activity of *T. harzianum* after 120 h.

#### Quantification of Lipase Activity

Given the lipolytic efficiency of *T. harzianum* and *G. candidum*, this step investigated the quantification of lipase activity by supplementing IN-BM and DEF-BM with the hydration solutions plus glucose or sucrose when desired (SN3SS, SN3SG, SN7SS, and SN7SG). In addition, the influence of the support degreasing process on enzyme activity was also evaluated. Initially, the titrimetric and spectrophotometric methods described above were compared. Although the analysis of the extracts using the two methods showed similar profiles in all conditions, both with *T. harzianum* and *G. candidum*, the best results were obtained using the titrimetric method, the results of which can be seen in Figure 4. The full results obtained by the two methods can be found in Tables S2.1 and S2.2 of the Supporting Information

**Figure 4:**
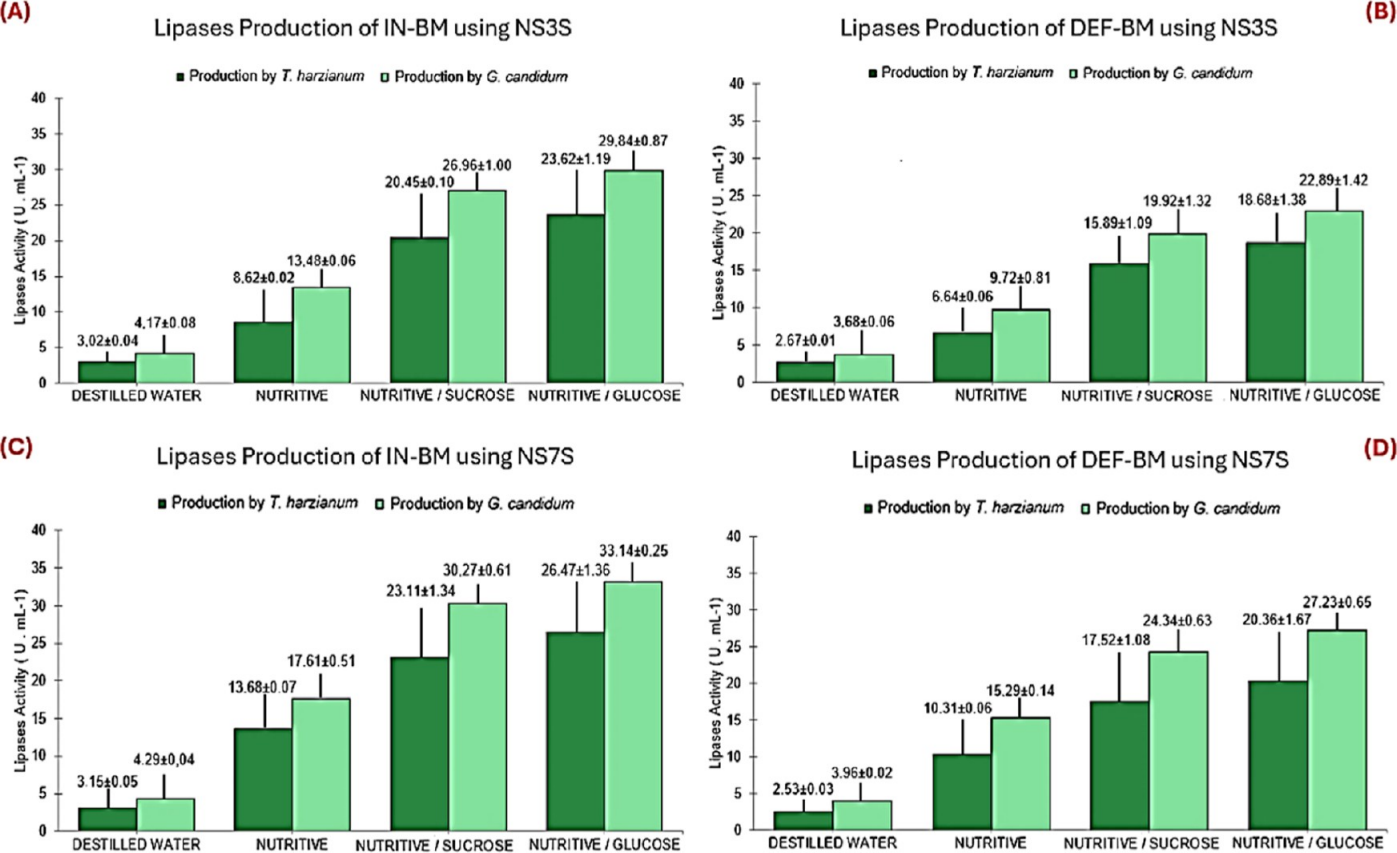
Lipase activity of *T. harzianum* and *G. candidum* in the SSF of babassu mesocarp after 120 h of fermentation. (A) SSF with IN-BM using hydration solutions with three salts. (B) SSF with DEF-BM using hydration solutions with three salts. (C) SSF with IN-BM using hydration solutions with seven salts. (D) SSF with DEF-BM using hydration solutions with seven salts. Analysis was carried out using the titrimetric method. Number of samples (*n* = 3).

Lipases are known as α/β hydrolases, with a catalytic site generally composed of a nucleophilic residue (serine), an acidic residue (aspartate or glutamate), and histidine.^45^ Some lipases have the catalytic site protected by a hydrophobic lid. In the presence of hydrophobic substrates, this lid shifts, changing its conformation and exposing the catalytic site to the substrate.^45,46^ This effect is called interfacial activation. However, interfacial activation is not necessarily related to interfacial activation since lipases from yeasts (*Candida antarctica* B), fungi such as *Rhizomucor miehei*, or even bacteria (*Pseudomonas aeruginosa* and *Burkholderia glumae*) can present “*Lid*” in their structures but do not undergo interfacial activation.^45^ In this sense, as both methods verified enzymatic activity, the lipases produced may not have a hydrophobic lid, or there may be no need for interfacial activation. Although the enzyme showed an affinity for both methods, the titrimetric method with arabic gum emulsion may have provided the best result, as arabic gum not only acts as a natural emulsifier, stabilizing emulsions by dispersing the lipid phase in the aqueous phase but can also reduce the interfacial tension between these phases, promoting a more significant interaction between the lipase and the lipids, resulting in more efficient hydrolysis.^47^

According to the previous figure, the highest lipase activity (U mL^–1^) was obtained in the IN-BM fermentation using *G. candidum* and the SN7SG solution (33.14 ± 0.25) (Figure 4D). This result was used as the basis for the calculations. It was considered 11.06% higher than that obtained with the SN3SG solution and 20.13% higher than the activity obtained with *T. harzianum* under the same conditions. As observed, the defatting process did not cause significant changes in the lipase production profile, demonstrating that the process is unnecessary for producing the enzyme with *T. harzianum* and *G. candidum*. It was also found that, under the conditions of this study, *G. candidum* was more lipolytic than *T. harzianum*.

To assess a possible cost reduction in the production process, the results obtained from fermenting *G. candidum* with SN7SS and SN7SG were also compared, with the latter being 9.48% higher. Ferreira (2017)^48^ obtained lipase activity equivalent to 22.9 U mL^–1^ after 48 h of fermentation using *G. candidum* NRRL Y-552 in FS supplemented with cottonseed oil, while Santos (2021),^49^ using the same strain of *G. candidum* as in the previous study and a medium supplemented with cottonseed oil, also obtained enzyme activity equivalent to 220.9 U mL^–1^ after 48 h of fermentation. Canseco-Pérez et al. (2018),^44^ using *T. harzianum* B13-1, obtained lipase activity equivalent to 20.5 U mL^–1^ after 8 days of SSF, while Toscano et al. (2013),^50^ using *T. harzianum* in wheat bran SSF, obtained activity equivalent to 14.3 U mL^–1^. Ülker et al. (2011)^51^ tested different types of carbon and nitrogen sources for the production of lipases by *T. harzianum* IDM14D and obtained, using glucose and peptone (0.24 U mL^–1^) of enzymatic activity after 7 days of SSF.

In both fermentations with *T. harzianum* and *G. candidum*, the supplementation of IN-BM and DEF-BM with NS3S and NS7S led to an increase in lipase activity compared to the control, in which activity was lower. Combined with a carbon source, this supplementation led to a significant increase in enzyme activity. This is mainly because these solutions contain micronutrients, which play a crucial role in the metabolism of microorganisms, mainly because they are required by the cellular machinery as cofactors for various processes.^30,52^ Although supplementing the hydration solution with glucose provided the best result, it was slightly higher than that obtained with sucrose supplementation, and there were no significant problems with its replacement. Because the strains did not produce amylases, it was necessary to supplement the biomass. However, in addition to producing enzymes, this bioprocess can also be an effective pretreatment for obtaining a starch-rich fraction for industrial applications.

Based on the experimental data shown in Figure 4, a statistical analysis of the data was carried out using Student’s paired *t*-test for the mean. This analysis was carried out to assess whether the supplementation of IN-BM and DEF-BM with NS3SG, NS3SS, NS7SG, and NS7SS shows significant differences in the production of lipases and laccases. These solutions were considered for the test because they showed the highest enzyme production rates. Table 2 shows the statistical analysis for two population samples (IN-BM and DEF-BM) containing two variables: the presence of 3 or 7 salts accompanied by sucrose or glucose (for more information, see Table S6 in the Supporting Information).

**Table 2:** Statistical Analysis of Fermentations Containing NS3SS, NS3SG, NS7SS e, NS7SG^a^

	IN-BM				DEF-BM			
statistical variable	NS3SG	NS3SS	NS7SG	NS7SS	NS3SG	NS3SS	NS7SG	NS7SS
	Lipase Activity—T. harzianum (U mL^–1^)							
mean	23.62^a^	20.45^b^	26.47^c^	23.11^d^	18.68^e^	15.89^f^	20.36^g^	17.52^g^
median	23.51	20.07	26.79	22.93	18.88	15.93	19.92	17.42
variance	1.41	0.95	1.85	0.80	1.91	1.19	2.80	1.17
standard deviation	1.19	0.98	1.36	1.34	1.38	1.09	1.67	1.08
	Lipase Activity**—**G. candidum (U mL^–1^)							
mean	29.84^h^	26.96^i^	33.14^j^	30.27^k^	22.89^l^	19.92^m^	27.23^n^	24.34^o^
median	29.82	26.76	33.10	30.17	22.83	19.67	26.98	24.26
variance	0.76	0.99	0,06	0,37	2.02	1.75	0.43	0.40
standard deviation	0.87	1.00	0.25	0.61	1.42	1.32	0.65	0.63

a Caption: IN-BM (in natura babassu mesocarp); DEF-BM (defatted babassu mesocarp); NS3S—nutrient solution with three salts; NS3SS—nutrient solution with three salts and sucrose; NS3SG—nutrient solution with three salts and glucose; NS7SS—nutrient solution with seven salts and sucrose; NS7SG—nutrient solution with seven salts and glucose. The letters following the numbers represent the statistical evaluation (Student’s *t*-test, 5% significance level). Equal letters mean that the samples are statistically equal, and different letters mean that the samples are statistically different. Number of samples (*n* = 3).

The quantitative analysis of the enzyme indicated that the fermentations containing NS3S and sugar as a carbon source (sucrose or glucose) provided the most relevant enzyme activities. The data obtained by the paired *t*-test resembles a normal distribution with equivalent variance and allows us to assess whether the results are the same or different. Thus, concerning lipase activity with *G. candidum* and *T. harzianum*, all the values were less than 0.05. They were, therefore, considered statistically different, and the null hypothesis was rejected, except for the results obtained when comparing fermentations with *T. harzianum* using DEF-BM and NS7SG and DEF-BM and NS7SS, for which the null hypothesis is accepted. No statistical difference is found between the two NS. Based on this analysis, the *G. candidum*, IN-BM, and NS7SG systems were considered the most suitable for lipase production.

#### Evaluation and Quantification of Laccase Activity

The quantification of laccase activity was carried out using the same extracts produced for the quantification of lipase, and the influence of hydration solutions on IN-BM and DEF-BM, as well as the supplementation of carbon sources, was evaluated. At this stage, the laccase quantification methods showed very similar profiles for both microorganisms under the conditions of this study. However, the ABTS oxidation results were more satisfactory, possibly indicating the enzyme’s greater affinity for this substrate. These results can be seen in the graphs in Figure 5 (more information can be found in Tables S2.3 and S2.4 of the Supporting Information).

**Figure 5:**
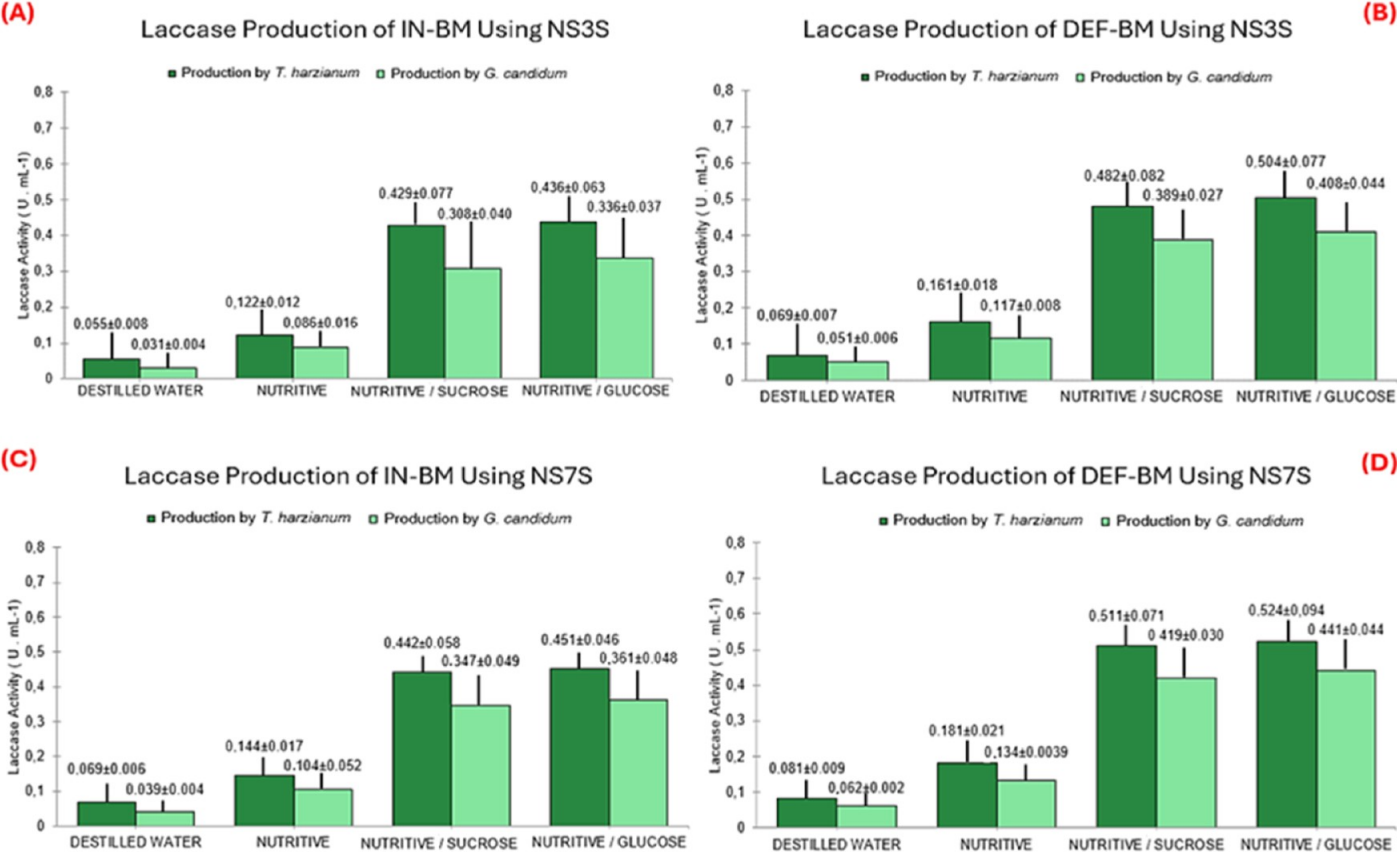
Laccase activity of *T. harzianum* and *G. candidum* in the SSF of babassu mesocarp after 120 h of fermentation. (A) SSF with IN-BM using hydration solutions with three salts. (B) SSF with DEF-BM using hydration solutions with three salts. (C) SSF with IN-BM using hydration solutions with seven salts. (D) SSF with DEF-BM using hydration solutions with seven salts. Analysis was carried out using the ABTS method. Number of samples (*n* = 3).

The more robust laccase activity results found by the ABTS method can be explained by the greater affinity of this substrate with the active site of laccases, making it possible to detect activity at even lower concentrations of the enzyme in the samples. In addition, ABTS is more stable over a wider pH range and can be advantageous under variable process reaction conditions.^53,54^ The highest laccase activity (U·mL^–1^) was obtained by fermenting DEF-BM using *T. harzianum* and the SN7SG solution (0.524 ± 0.094) (Figure 5D), which was 3.97% higher than that obtained with the SN3SG solution and 18.82% higher than that obtained with *G. candidum*. For both fungi, the difference in lipid concentration in IN-BM and DEF-BM did not provide a significant difference in the final laccase activity. The economic context was also considered at this stage, so the activities obtained from fermenting *T. harzianum* with SN7SS and SN7SG were compared, with the latter being only 2.54% higher. This result shows that laccase production can be achieved using the least number of salts and the most economical carbon source, sucrose.

The maximum activity obtained by *T. harzianum* can be considered low compared to that observed in the study by Bagewadi et al. (2017)^53^ (162.5 U·mL^–1^) and Sadhasivam et al. (2008)^54^ (4.36 U·mL^–1^), but it was close to that found in the study by Elsayed et al. (2023)^55^ for LacA and LacB (0.603 and 0.182 U·mL respectively). The laccase activity obtained from *G. candidum* through SSF with agro-industrial waste is being reported for the first time in the literature. The study by Suju and Nair (2016)^56^ was the only work to report the production of laccase by *G. candidum* and, according to the authors, at the end of the fermentation, it was possible to achieve (0.160 U·mL^–1^) of enzymatic activity with nutrient medium and (0.253 U·mL^–1^) with optimized medium. These results were close to those obtained in the present study; however, the above data refer to submerged fermentation.

The statistical analysis of laccase production (Table 3) used the same criteria and methods as the previous study and was based on the results shown in Figure 5. More information on the Supporting Information can be found in Table S6.

**Table 3:** Statistical Analysis of Fermentations Containing NS3SS, NS3SG, NS7SS e, NS7SG^a^

	IN-BM				DEF-BM			
	NS3SG	NS3SS	NS7SG	NS7SS	NS3SG	NS3SS	NS7SG	NS7SS
	Laccase—T. harzianum							
mean	0.436^a^	0.429^a^	0.451^a^	0.442^a^	0.504^a^	0.482^a^	0.524^a^	0.511^a^
median	0.429	0.408	0.439	0.440	0.492	0.457	0.534	0.522
variance	0.004	0.006	0.002	0.003	0.006	0.007	0.009	0.005
standard deviation	0.063	0.077	0.046	0.058	2.47	2.19	0.094	0.071
	Laccase—G. candidum							
mean	0.336^a^	0.308^a^	0.361^a^	0.347^a^	0.408^a^	0.389^a^	0.441^a^	0.419^a^
median	0.339	0.298	0.339	0.327	0.397	0.397	0.442	0.419
variance	0.001	0.002	0.002	0.002	0.002	0.001	0.002	0.001
standard deviation	0.037	0.040	0.048	0.049	0.044	0.027	0.044	0.030

a Caption: IN-BM (in natura babassu mesocarp); DEF-BM (defatted babassu mesocarp); NS3S—nutrient solution with three salts; NS3SS—nutrient solution with three salts and sucrose; NS3SG—nutrient solution with three salts and glucose; NS7SS—nutrient solution with seven salts and sucrose; NS7SG—nutrient solution with seven salts and glucose. The letters following the numbers represent the statistical evaluation (Student’s *t*-test, 5% significance level). Equal letters mean that the samples are statistically equal, and different letters mean that the samples are statistically different. Number of samples (*n* = 3).

According to the statistical treatment, all the comparisons showed a result greater than 0.05 for both microorganisms, so they were considered statistically equal, and the null hypothesis was accepted. Thus, the system is considered most suitable for laccase production was formed by *T. harzianum*, DEF-BM, and NS7SG, which was investigated with the best lipase production system (obtained with *G. candidum*) in the next step.

Overall, as important observations, it was clear that the system needs the addition of nutrient solutions to increase the productivity of both lipase and laccase. However, the addition of sucrose was just as efficient as the addition of glucose, and could be an indication of lower cost. Thus, the addition of glucose or sucrose was able to favor the production of these enzymes, since these sugars were assimilated by the microorganisms as an external carbon source. Hydration solutions contain micronutrients, such as zinc, sulfur, magnesium, manganese and yeast extract, for example, which play an extremely important role in the metabolism of microorganisms. These micronutrients can have a positive or negative effect on the metabolism of microorganisms. Thus, NS7S may have had a positive effect on lipase production, but some components of this solution may have had a negative effect during fermentation, impairing laccase production compared to the effects of NS3S.

#### Lipase and Laccase Production Kinetics

Considering the results previously obtained, lipase and laccase production was evaluated over 192 h of cultivation to determine the maximum production peak. Samples from independent cultures were analyzed every 24 h. The kinetic curves of lipase and laccase activity are shown in Figures 6 and 7. More information on the Supporting Information can be found in Table S5.

**Figure 6:**
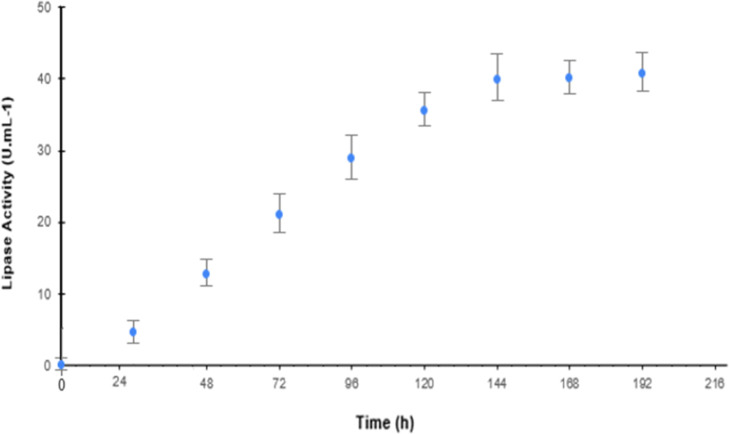
Kinetics of lipase production of *G. candidum* in IN-BM supplemented with SN7SG. Enzymatic activity was assessed over 192 h using a titrimetric method with gum Arabic emulsion as a substrate. Standard deviation (SD = 1); number of samples (*n* = 3). Cultivation conditions: temperature 30 °C, humidity 90%, 2.0 mL of hydration solution (pH 7.0), and 1.0 mL of spore suspension containing 1 × 10^5^ spores/mL.

**Figure 7:**
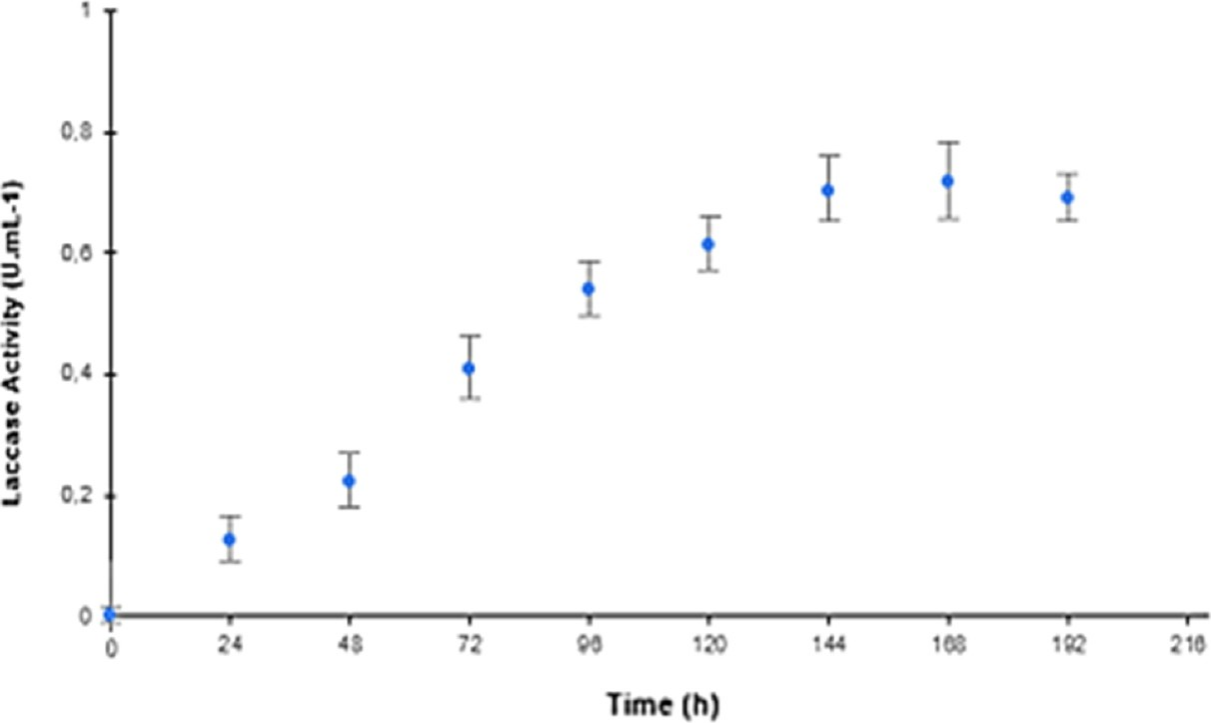
Kinetics of laccase production by *T. harzianum* in DEF-BM supplemented with SN7SG. Enzymatic activity was evaluated for 192 h using the ABTS method. Standard deviation (SD = 1); Number of samples (*n* = 3). Cultivation conditions: temperature 30 °C, humidity 90%, 2.0 mL of hydration solution (pH 7.0), and 1.0 mL of spore suspension containing 1 × 10^5^ spores/mL.

The kinetic analysis showed that the two enzymes had similar production profiles, with increases in activity after 72 h, reaching maximum productivity after 144 h of cultivation.

The maximum production achieved for lipase production (U mL^–1^) was (39.92 ± 2.04), while the maximum laccase production (U mL^–1^) was equivalent to (0.702 ± 0.089). These results correspond to an increase of 20.5% in lipase activity and 33.97% in laccase activity when compared to the results obtained in the fermentation with 120 h of cultivation and reflect the importance of monitoring production kinetics to determine the ideal cultivation time, intending to reduce costs and the yield of the final product.

### Protein Profile of the Enzyme Extracts (SDS-PAGE)

Protein quantification in the enzyme extracts showed that *T. harzianum* had 102.12 mg·L^–1^ of protein in IN-BM and 126.27 mg·L^–1^ in DEF-BM. The analysis for *G. candidum* showed 157.24 mg·L^–1^ of protein using IN-BM and 129.93 mg·L^–1^ using DEF-BM.

Preliminary gel electrophoresis results show that the extract obtained from the fermentation of DEF-BM with *T. harzianum* has four bands with molar masses of 150 kDa, 100 kDa (discrete), 50 kDa, and 37 kDa. The extract obtained from fermentation with IN-BM has two bands with molar masses between 100 kDa and 50 kDa and between 37 kDa and 25 kDa (Figure 8). The analysis was carried out by comparing the bands obtained in the extract with the bands of the standard, which range from 250 to 10 kDa. These results were also compared with the commercial lipase from *Thermomyces lanuginosus*, *Lipozyme* TL 100L (TLL), which showed a single homogeneous band between 50 kDa and 37 kDa, similar to the one observed in the DEF-BM extract, suggesting that this 50 kDa band in the extract may be a lipase. A comparison was also made with the commercial laccase from *Aspergillus niger*, which showed a single homogeneous band between 37 kDa and 25 kDa.

**Figure 8:**
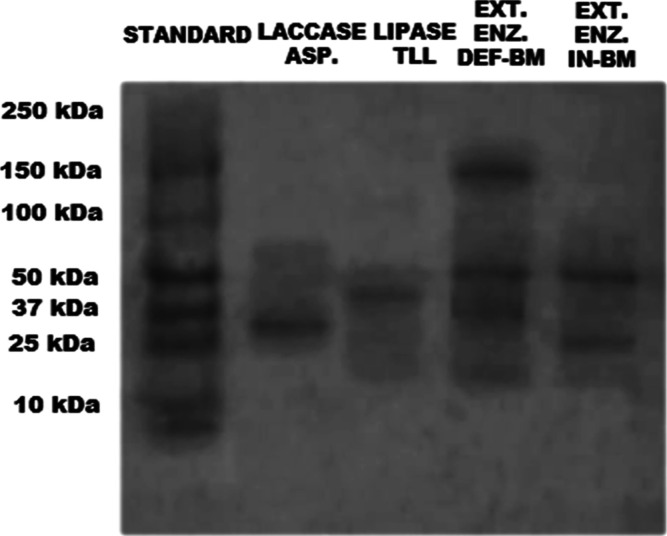
Gel electrophoresis (SDS-PAGE) of the crude enzyme extract obtained from the fermentation of IN-BM and DEF-BM with *T. harzianum*. Lacase ASP (laccase from *Aspergillus niger*), lipase TLL (Lipase from *Thermomyces lanuginosus*), Extr. Enz. IN-BM (enzymatic extract obtained from the fermentation of babassu mesocarp in natura), Enz. Extr. DEF-BM (enzymatic extract obtained from the fermentation of defatted babassu mesocarp).

According to Souza (2023),^57^ TLL has a molecular weight of 31.7 kDa so that the lipase may have appeared on the gel early in this work. Another hypothesis for the bands obtained in the extract is that the 50 kDa band and the 37 kDa band may be lipases since, according to Jorge et al. (2018)^58^ and Vaz (2010),^59^ *T. harzianum* can produce lipase isoforms. In their study, two isoforms of the enzyme were observed, with approximately 56 kDa and 44 kDa (Lip1 and Lip2), respectively. There is also the possibility that the 37 kDa band and the band between 50 kDa and 100 kDa belong to laccase isoforms since, according to Elsaved et al. (2023),^55^ LacB from *T. harzianum* has a molecular weight of approximately 48 kDa and, according to Sadhasivam et al. (2008),^54^ this microorganism can also secrete laccase with a molecular weight of approximately 79 kDa. Polyacrylamide gel analysis of the IN-BM extract showed two homogeneous bands with molecular weights of 50 kDa and approximately 25 kDa. This suggests that these bands belong to an isoform of lipase and laccase.

Preliminary analysis of the extract obtained from fermentations of IN-BM with *G. candidum* showed four bands, two of which had molar masses close to 100 kDa and close to 50 kDa, with 37 kDa and 25 kDa (Figure 9). As in the previous analysis, the enzymes observed in the extracts produced from *G. candidum* were compared with TLL and laccase from *A. niger*, suggesting that the microorganism secretes different isoforms of the lipase and laccase enzymes when compared to *T. harzianum*. The bands corresponding to approximately 50 kDa and 37 kDa suggest lipase isoforms. These results were relatively close to those observed in the study by Junqueira (2014),^60^ in which the author obtained the enzyme in the range of approximately 60 kDa, and to those observed in the study by Morais (2016),^61^ in which the author obtained the enzyme in the molecular weight range of 38.3 kDa using a medium containing soy molasses. As for the extract obtained with DEF-BM, a band was observed in the molecular weight range between 50 kDa and 37 kDa, suggestive of the laccase enzyme (when compared to the commercial laccase from *A. niger*), and a band between 50 kDa and 100 kDa, suggestive of a lipase isoform.

**Figure 9:**
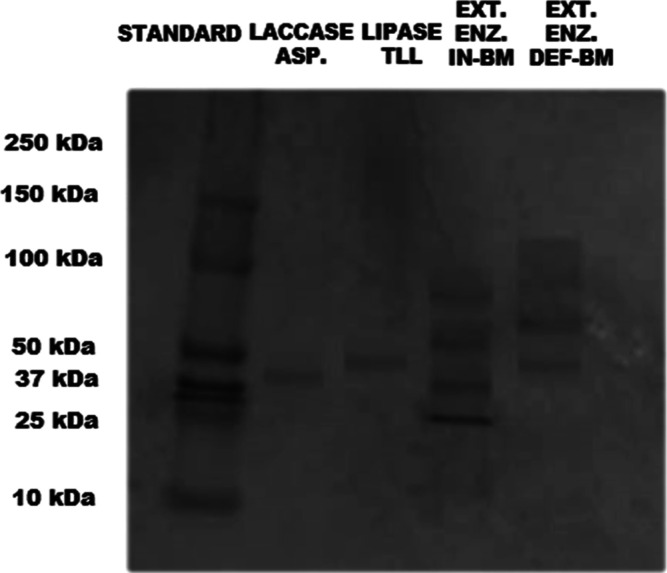
Gel electrophoresis (SDS-PAGE) of the crude enzyme extract obtained from the fermentation of IN-BM and DEF-BM with *G. candidum*. Lacase ASP (laccase from *Aspergillus niger*), lipase TLL (Lipase from *Thermomyces lanuginosus*), Extr. Enz. IN-BM (enzymatic extract obtained from the fermentation of babassu mesocarp in natura), Enz. Extr. DEF-BM (enzymatic extract obtained from the fermentation of defatted babassu mesocarp).

Due to the lack of studies characterizing *G. candidum* laccase, it was impossible to measure the enzyme’s possible molecular weight. However, when comparing the *T. harzianum* laccase analyzed in this work and the *G. candidum* laccase, both occur in similar molecular weight ranges, and the laccase enzyme activity results reinforce the presence of this molecule in the extracts. Given the preliminary nature of this study, a more detailed analysis of these molecules could help to define the molecular weights and the precise identification of each enzyme.

### Analysis of the Surface of the Fermented Supports—Scanning Electron Microscopy and Analytical Magnifier

The SEM was used to analyze the surface of the fermented supports (Figure 10). Although the growth of the microorganisms was observed in most of the fermentations, for this analysis, the fermentations that showed the best development after 7 days of cultivation were considered, and the fermentations carried out with the NS7SS hydration solution were chosen. The analysis showed that the microorganisms grew in the fermentations containing this hydration solution, forming a uniform cover over the waste.

**Figure 10:**
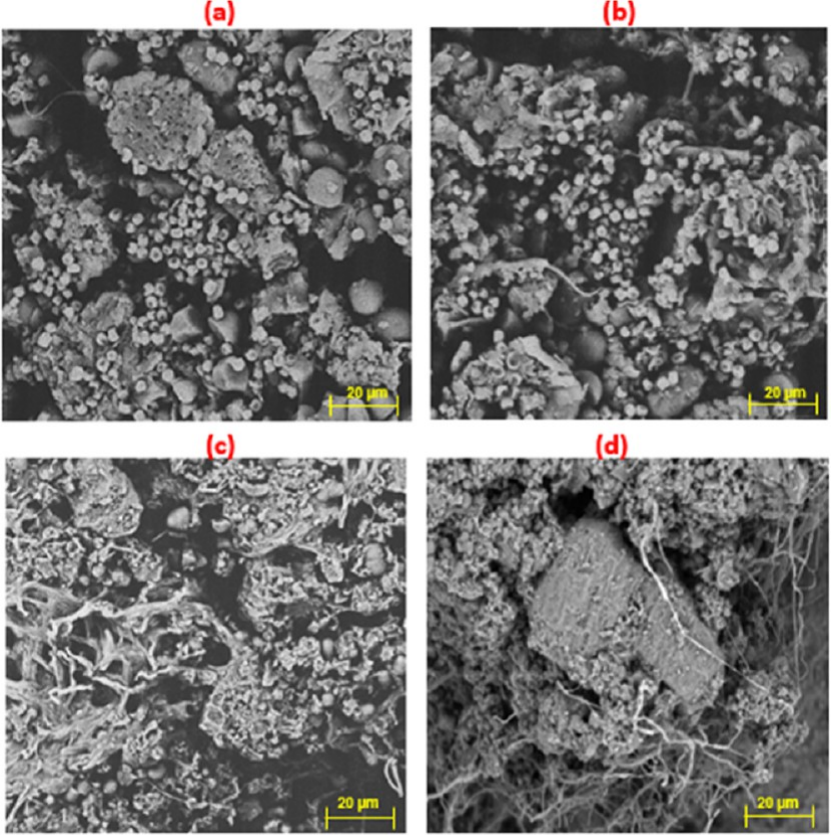
Scanning electron microscopy in natura and treated support fermented with *T. harzianum* and *G. candidum*, using NS7SG solution. (a) IN-BM fermented with *T. harzianum*; (b) DEF-BM fermented with *T. harzianum*; (c) IN-BM fermented with *G. candidum*; (d) DEF-BM fermented with *G. candidum*.

The development of *T. harzianum* was visually assessed throughout the fermentation, and spore formation was observed from the third day of fermentation on DEF-BM and the fourth day on IN-BM and was quite significant at the end of the fermentations with NS3SS, NS3SG, NS7SS and NS7SG on both supports. As for the development of *G. candidum*, spores were formed from the fourth day onward on both supports. It was also more expressive on the seventh day of fermentation but more discrete than the sporulation shown by *T. harzianum*. Because of these observations, the fermented supports analyzed by SEM were also investigated using an analytical magnifying glass (Figure 11). The results indicate that the BM impregnated with the nutrient solution was satisfactory as a support, as both *T. harzianum* and *G. candidum* developed on this biomass, forming vegetative structures and spreading and entering the gaps in the support.

**Figure 11:**
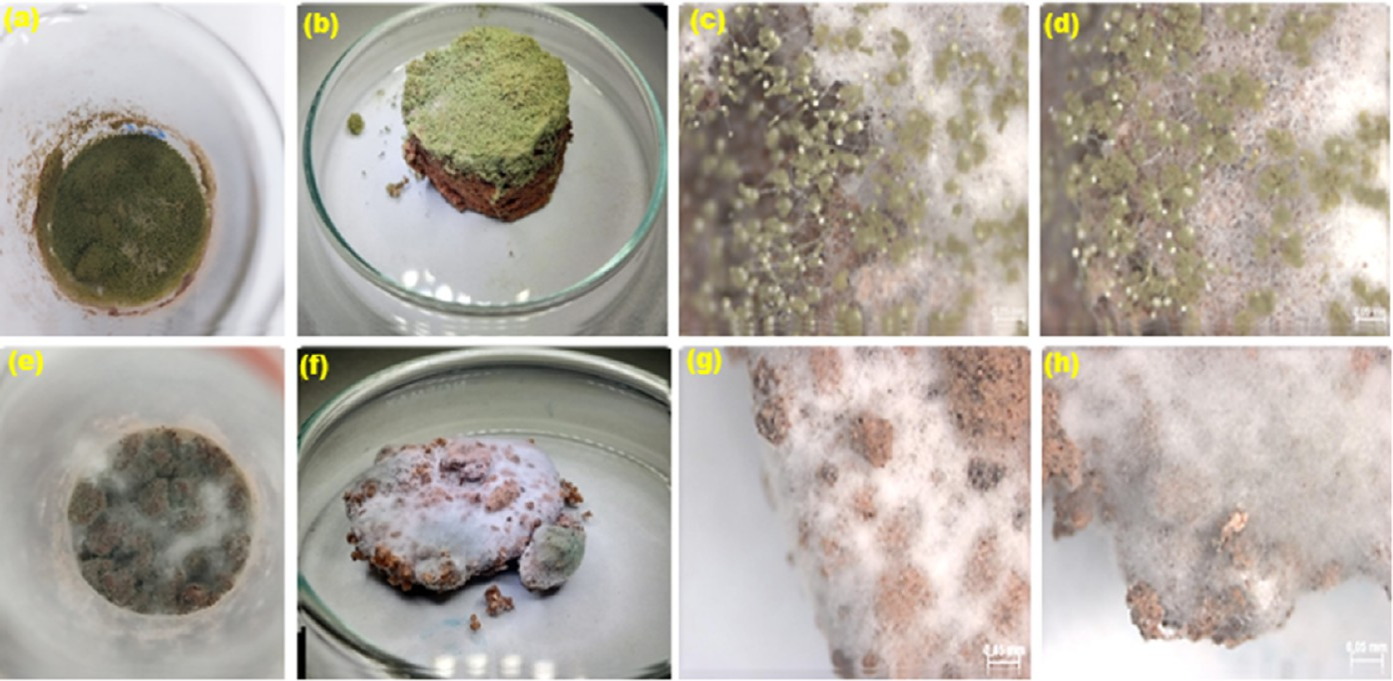
Surface analysis of the in natura and treated fermented support, fermented with *T. harzianum* and *G. candidum*, using NS7SG solution. (a) Internal image of the fermentation with *T. harzianum*; (b) visual aspect of the support fermented with *T. harzianum*; (c,d) development of the vegetative structures of *T. harzianum* in the greased (in natura) and degreased support, respectively. (e) Internal image of the fermentation with *G. candidum*; (f) visual aspect of the support fermented with *G. candidum*; (g,h) development of the vegetative structures of *G. candidum* on the greased and defatted support, respectively.

In order to check if microorganisms were able to degrade the starch granules of supports, a surface analysis was performed. The micrographs presented in Figure 12 show that the starch granules of the supports were minimally degraded during fermentation, with these structures totally or partially intact, since some of the granules showed only small cracks (Figure 12a,b). This profile was observed both in the IN-BM and DEF-BM supports fermented with *T. harzianum* (Figure 12c,d) and in the IN-BM and DEF-BM supports fermented with *G. candidum* (Figure 12e,f). These images corroborate the negative result for amylase activity found for both strains, demonstrating the need for supplementation and the possibility of pretreatment to release starch since they could metabolize lipids and lignin.

**Figure 12:**
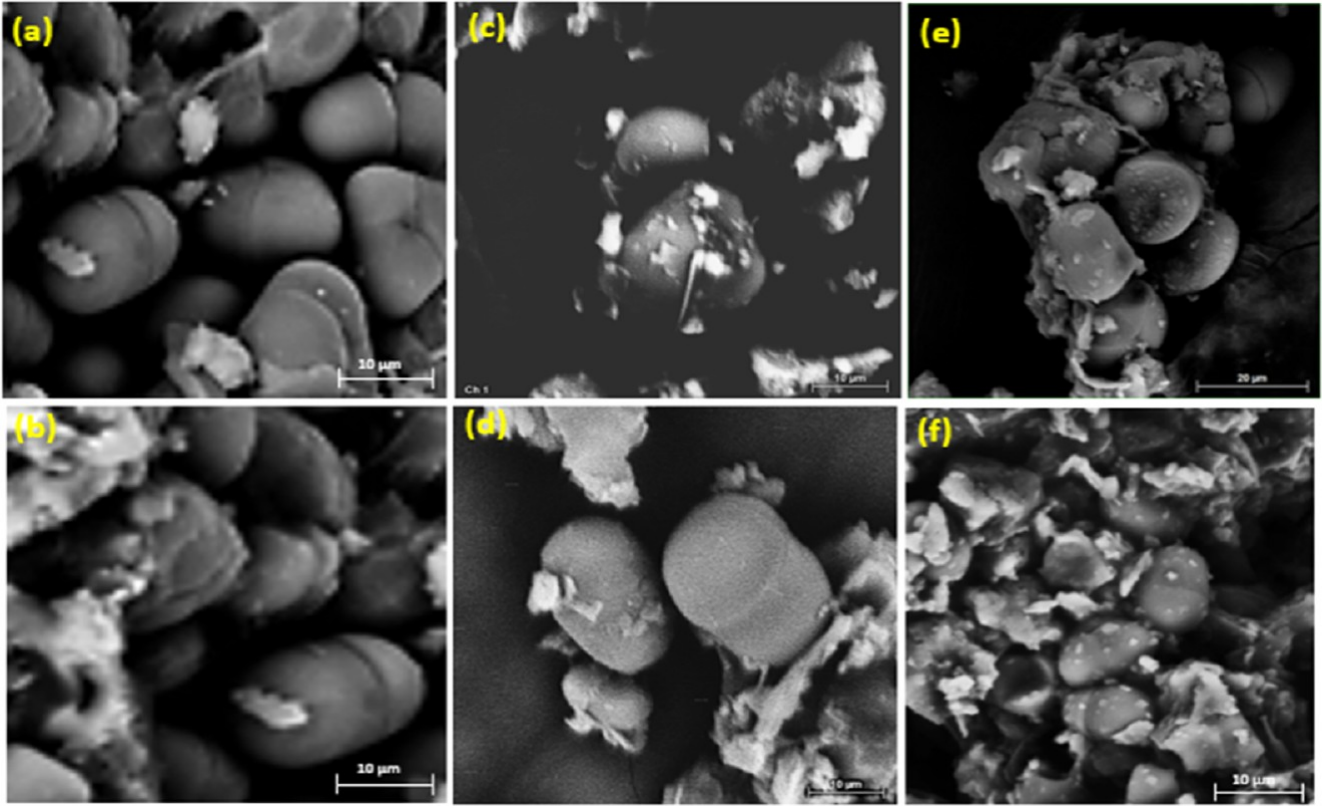
Micrograph of the starch granules of the supports obtained by SEM (a) IN-BM in the absence of microorganisms (control); (b) DEF-BM in the absence of microorganisms (control); (c) IN-BM fermented with *T. harzianum*; (d) DEF-BM fermented with *T. harzianum*; (e) IN-BM fermented with *G. candidum*; (f) DEF-BM fermented with *G. candidum*.

## Conclusions

In this work, it was possible to demonstrate that BM is a versatile agro-industrial waste used to produce lipases and laccases by *G. candidum* and *T. harzianum*, where the best conditions for each enzyme could be modeled. In addition, the ability of *G. candidum* to produce laccases is reported for the first time, demonstrating the high biotechnological capacity of this species. With the fungi studied, the hydration solution containing three salts and sucrose was the best condition for obtaining maximum enzyme activity. In this way, our bioprocess could be a cheap and sustainable option to valorize BM through SSF to obtain enzymes of great industrial value and also carry out a biological pretreatment, degrading the lignin fraction, the lipid fraction and releasing the starch fraction for industrial applications. SSF is a simple method, but it presents a high risk of contamination, does not allow efficient temperature control and can present mass transfer problems. However, it is a practical and viable tool for the valorization of BM. Further studies need to be carried out to investigate the feasibility of scale-up and economic studies.

## Supplementary Material



## Notes

Item S1: Determination of protein concentration; S1.1: Protein concentration of enzyme extracts; Item S2: Enzymatic activity of microorganisms in babassu mesocarp; S2.1: Lipase activity of *T. harzianum* using *p*-nitrophenol and using the titrimetric method; S2.2: Lipase activity of G. candidum using *p*-nitrophenol and using the titrimetric method; S2.3: Laccase activity of *T. harzianum* using ABTS method and using guaiacol; S2.4: Laccase activity of *G. candidum* using ABTS method and using guaiacol; Item S3: Statistical test (PDF)
