# Supplementary material for: Valorization of Residual Babassu Mesocarp To Obtain Lipases and Laccases by Solid-State Fermentation
Source: ACS Omega. 2025 Jun 30;10(27):29665–79. doi: 10.1021/acsomega.5c03496 (PMC12268442; doi:10.1021/acsomega.5c03496)
Supplement: Supplementary file 1 [file ao5c03496_si_002.pdf]

Valorization of Residual Babassu Mesocarp to Obtain Lipases and Laccases by Solid-State Fermentation

Tamires N. dos Anjos [1], Selma G. F. Leite [1], Ivana C. R. Leal [2] and Ivaldo Itabaiana Jr [1]*

[1]Department of Biochemical Engineering, School of Chemistry, Federal University of Rio de Janeiro, Rio de Janeiro 21941-909, Brazil.

[2]Department of Natural Products and Food, Faculty of Pharmacy, Federal University of Rio de Janeiro, Rio de Janeiro 21941-902, Brazil.

Supplementary Material

SUMMARY

S1. Determination of protein concentration………………………………………………………. **S2**

S1.1. Protein concentration of enzyme extracts…………………………………………………... **S2**

S2. Enzymatic activity of microorganisms in babassu mesocarp…………………………………… **S2**

S2.1. Lipase activity of *Trichoderma harzianum* using *p*-nitrophenol and using the titrimetric method……………………………………………………………………………………………. **S3**

S2.2. Lipase activity of *Geotrichum candidum* using *p*-nitrophenol and using the titrimetric method…………………………………………………………………………………………….. **S4**

S2.3. Laccase activity of *Trichoderma harzianum* using ABTS method and using guaiacol………. **S4**

S2.4. Laccase activity of *Geotrichum candidum* using ABTS method and using guaiacol………… **S5**

S3. Statistical test……………………………………………………………………………………. **S6**

S1. Determination of Protein Concentration

S1.1. Calibration curve for protein concentration determination

The calibration curve for determining the concentration of proteins in the enzyme extracts was drawn up using the Bradford method. Sigma's bovine serum albumin (BSA) was used as a standard and prepared with sodium phosphate buffer (0.025 M, pH 7.0) at concentrations ranging from 10 to 100 mg/mL of BSA. The Bradford reagent was prepared using 0.1 g of Coomassie Brilliant Blue G-250 dye (BioRad protein assay dye reagent concentrate) dissolved in 0.05 L of 95% ethyl alcohol, 0.1 L of 85% orthophosphoric acid and the volume completed to 1.0 L with ultrapure water. In a 96-well plate, 20 µL of sample was incubated with 180 µL of Bradford reagent, keeping the system stirring for 10 minutes at room temperature. The absorbance was determined at 595 nm using a microplate reader (PowerWave XS from Biotek) and the linear regression equation of the calibration curve (Fig. S1), correlating the absorbance values to the concentration of proteins dissolved in the solutions, was used to determine the concentration of proteins in the enzymatic extracts of the fermentations.

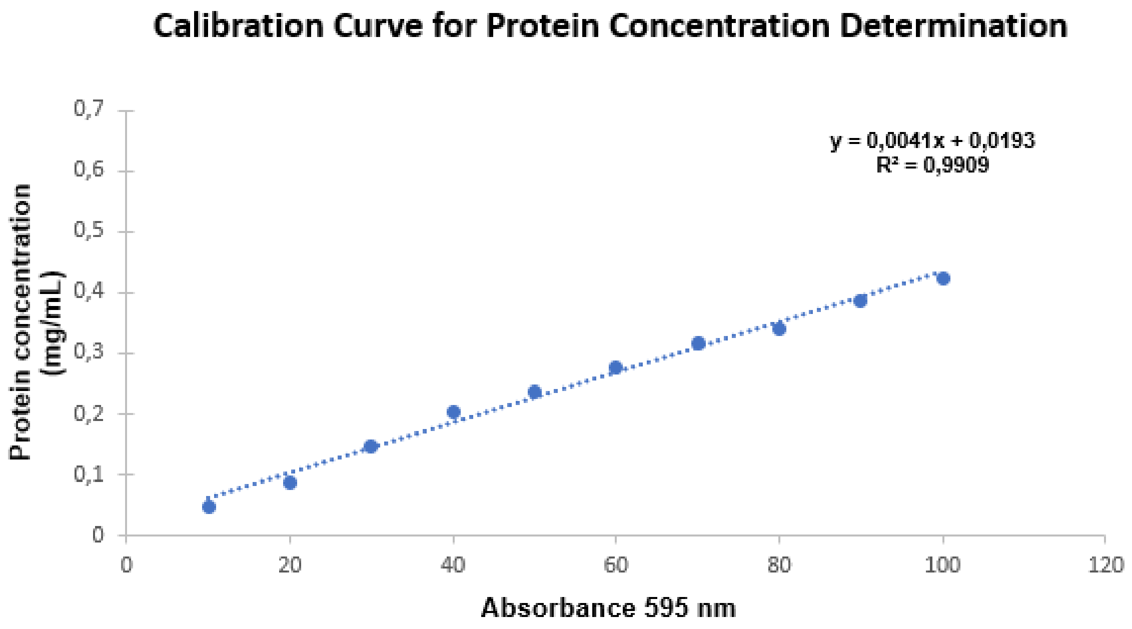

Fig. S1 Calibration curve for determining the concentration of proteins in enzyme extracts using the Bradford method

S2. Enzymatic activity of microorganisms in babassu mesocarp

The lipase activity of the microorganisms (Table S1 and S2) was analyzed by titrimetric method using the hydrolysis of an emulsion containing: olive oil, gum arabic (5% m/v) and sodium phosphate buffer (100 mM and ph 7.0). Quantification was carried out using a Mettler Toledo Compact G20S automatic titrator. The analysis was also carried out using p-nitrophenol, in order to evaluate the best analysis method. A 2.5 mM solution of ρ-nitrophenyl laurate (*ρ*-NFL) was prepared, consisting of 100 mL dimethylsulfoxide (DMSO):acetonitrile (1:1) solution and 0.08 g of *ρ*-NFL, which was used as

the reaction substrate. The absorbance of the chromophore product formed (ρ-nitro phenol) was read on a Bel Photonics VM5 spectrophotometer at 412 nm at 30-second intervals for 5 minutes.

As for laccase activity (Table S3 and S4), this was analyzed through the oxidation of ABTS (2,2'-azino-bis-3-ethylbenzothiazoline-6-sulfonic acid). The reaction was carried out using 1.7 mL of 100 mM sodium acetate buffer (pH 5.5), 100 µL of enzyme extract and 200 µL of 10 mM ABTS solution. This system was kept in a water bath at 30°C for 5 minutes and, after this process, the absorbance of the samples was read in a Bel Photonics VM5 spectrophotometer at 420 nm, making sure that the reaction time did not exceed 5 minutes. The enzyme was also quantified using guaiacol as a substrate. The reaction was carried out using a mixture of 100 µL of sample, 100 µL of 10 mM guaiacol and 800 µL of 10 mM sodium acetate buffer, which was incubated in a water bath at 30°C for 15 minutes. The absorbance of the chromogen generated in the reaction was read on a Bel Photonics VM5 spectrophotometer at 450 nm.

S2.1. Lipase activity of *Trichoderma harzianum* using *p*-nitrophenol and using the titulometric method

Table S1 Lipase activity in babassu mesocarp fermented with *T. harzianum*, using a titulometric method and *p*-nitro phenol as a substrate

Lipase Activity (U.mL^{-1})								
Type of support	Methods	Hydration Solution						
		3 salts				7 salts		
		NS	NSS	NSG	DW	NS	NSS	NSG
IN-BM	Titulometric	8.62± 0.02	20.45± 0.10	23.62± 1.19	3.02± 0.04	13.68± 0.07	23.11± 1.34	26.47± 1.36
	p-nitro phenol	4.96± 0.03	15.87± 0.05	18.74± 1.03	1.88± 0.02	9.52± 0.10	17.94± 1.02	21.96± 0.82
DEF-BM	Titulometric	6.64± 0.06	15.89± 1.09	18.68± 1.38	2.67± 0.01	10.31± 0.06	17.52± 1.08	20.36± 1.67
	p-nitro phenol	3.94± 0.09	11.29± 0.07	13.42± 0.88	1.02± 0.09	5.88± 0.13	14.11± 0,96	16.18± 1,21

Caption: IN-BM (in natura babassu mesocarp); DEF-BM (defatted babassu mesocarp); NS - (nutrient solution with 3 salts (NS3S) – composition of the nutriente solution (mg/g of support): $(NH4)_2SO_4$, 0.943, KH_2PO_4, 1.0, $MgSO_4·7H_2O$, 5.0; NSS - nutrient solution with 3 salts and sucrose (NS3SS) - composition of the nutriente solution (mg/g of support): $(NH4)_2SO_4$, 0.943, KH_2PO_4, 1.0, $MgSO_4·7H_2O$, 5.0; sucrose, 1,0; NSG - nutrient solution with 3 salts and glucose (NS3SG) - composition of the nutriente solution (mg/g of support): $(NH4)_2SO_4$, 0.943, KH_2PO_4, 1.0, $MgSO_4·7H_2O$, 5.0; glucose, 1,0; DW - hydration solution (distilled water); NS - nutrient solution with 7 salts (NS7S) - Composition of the nutrient solution (mg/g of support): $(NH4)_2SO_4$, 0.943; yeast extract 1.0; $MgSO_4·7H_2O$, 5; KH_2PO_4, 1.0; KCl, 0.5; $CaCl_2·2H_2O$, 0.008; $FeSO_4·7H_2O$, 0.01; $ZnSO_4·7H_2O$, 0.001; NSS - nutrient solution with 7 salts and

sucrose (NS7SS) - Composition of the nutrient solution (mg/g of support): $(NH4)_2SO_4$, 0.943; yeast extract 1.0; $MgSO_4 \cdot 7H2O$, 5; KH_2PO_4, 1.0; KCl, 0.5; $CaCl_2 \cdot 2H_2O$, 0.008; $FeSO_4 \cdot 7H_2O$, 0.01; $ZnSO_4 \cdot 7H_2O$, 0.001; sucrose, 1,0. NSG - nutrient solution with 7 salts and glucose (NS7SG) Composition of the nutrient solution (mg/g of support): $(NH4)_2SO_4$, 0.943; yeast extract 1.0; $MgSO_4 \cdot 7H_2O$, 5; KH_2PO_4, 1.0; KCl, 0.5; $CaCl_2 \cdot 2H_2O$, 0.008; $FeSO_4 \cdot 7H_2O$, 0.01; $ZnSO_4 \cdot 7H_2O$, 0.001; glucose, 1,0.

S.2.2. Lipase activity of *Geotrichum candidum* using *p*-nitrophenol and using the titulometric method

Table S2 - Lipase activity in babassu mesocarp fermented with *G. candidum*, using a titulometric method and *p*-nitro phenol as a substrate.

Lipase Activity (U.mL^{-1})								
Type of support	Methods	Hydration Solution						
		3 salts				7 salts		
		NS	NSS	NSG	DW	NS	NSS	NSG
**IN-BM**	Titulometric	13.48± 0.06	26.96± 1.00	29.84± 0.87	4.29± 0.04	17.61± 0.51	30.27± 0.61	33.14± 0.25
	p-nitro phenol	9.72± 0.32	22.14± 0.72	25.32± 0.84	2.11± 0.06	14.02± 0.39	26.04± 0.65	28.88± 0.57
**DEF-BM**	Titulometric	9.72± 0.81	19.92± 1.32	22.89± 1.42	3.96± 0.02	15.29± 0.14	24.34± 0.63	27.23± 0.65
	p-nitro phenol	5.04± 0.28	15.19± 0.54	17.96± 0.27	2.01± 0.03	11.06± 0.81	19.97± 0.22	22.95± 0.53

Caption: IN-BM (in natura babassu mesocarp); DEF-BM (defatted babassu mesocarp); NS - (nutrient solution with 3 salts (NS3S) – composition of the nutriente solution (mg/g of support): $(NH4)_2SO_4$, 0.943, KH_2PO_4, 1.0, $MgSO_4 \cdot 7H_2O$, 5.0; NSS - nutrient solution with 3 salts and sucrose (NS3SS) - composition of the nutriente solution (mg/g of support): $(NH4)_2SO_4$, 0.943, KH_2PO_4, 1.0, $MgSO_4 \cdot 7H_2O$, 5.0; sucrose, 1,0; NSG - nutrient solution with 3 salts and glucose (NS3SG) - composition of the nutriente solution (mg/g of support): $(NH4)_2SO_4$, 0.943, KH_2PO_4, 1.0, $MgSO_4 \cdot 7H_2O$, 5.0; glucose, 1,0; DW - hydration solution (distilled water); NS - nutrient solution with 7 salts (NS7S) - Composition of the nutrient solution (mg/g of support): $(NH4)_2SO_4$, 0.943; yeast extract 1.0; $MgSO_4 \cdot 7H_2O$, 5; KH_2PO_4, 1.0; KCl, 0.5; $CaCl_2 \cdot 2H_2O$, 0.008; $FeSO_4 \cdot 7H_2O$, 0.01; $ZnSO_4 \cdot 7H_2O$, 0.001; NSS - nutrient solution with 7 salts and sucrose (NS7SS) - Composition of the nutrient solution (mg/g of support): $(NH4)_2SO_4$, 0.943; yeast extract 1.0; $MgSO_4 \cdot 7H2O$, 5; KH_2PO_4, 1.0; KCl, 0.5; $CaCl_2 \cdot 2H_2O$, 0.008; $FeSO_4 \cdot 7H_2O$, 0.01; $ZnSO_4 \cdot 7H_2O$, 0.001; sucrose, 1,0. NSG - nutrient solution with 7 salts and glucose (NS7SG) Composition of the nutrient solution (mg/g of support): $(NH4)_2SO_4$, 0.943; yeast extract 1.0; $MgSO_4 \cdot 7H_2O$, 5; KH_2PO_4, 1.0; KCl, 0.5; $CaCl_2 \cdot 2H_2O$, 0.008; $FeSO_4 \cdot 7H_2O$, 0.01; $ZnSO_4 \cdot 7H_2O$, 0.001; glucose, 1,0.

S2.3. Laccase activity of *Trichoderma harzianum* using ABTS method and using guaiacol

Table S3 – Laccase activity in babassu mesocarp fermented with *T. harzianum*, using ABTS and guaiacol as substrate.

Laccase Activity (U.mL^{-1})								
Type of support	Substrate	Hydration Solution						
		3 salts			DW	7 salts		
		NS	NSS	NSG		NS	NSS	NSG
**IN-BM**	ABTS	0.122± 0.012	0.429± 0.077	0.436± 0.063	0.069± 0.006	0.144± 0.017	0.442± 0.058	0.451± 0.046
	Guaiacol	0.093± 0.016	0.316± 0.028	0.323± 0.034	0.056± 0.002	0.116± 0.015	0.329± 0.049	0.346± 0.033
**DEF-BM**	ABTS	0.161± 0.018	0.482± 0.082	0.504± 0.077	0.081± 0.009	0.181± 0.021	0.511± 0.071	0.524± 0.094
	Guaiacol	0.136± 0.011	0.378± 0.075	0.401± 0.088	0.067± 0.011	0.152± 0.013	0.397± 0.064	0.413± 0.077

Caption: IN-BM (in natura babassu mesocarp); DEF-BM (defatted babassu mesocarp); NS - (nutrient solution with 3 salts (NS3S) – composition of the nutriente solution (mg/g of support): (NH4)2SO4, 0.943, KH2PO4, 1.0, MgSO4·7H2O, 5.0; NSS - nutrient solution with 3 salts and sucrose (NS3SS) - composition of the nutriente solution (mg/g of support): (NH4)2SO4, 0.943, KH2PO4, 1.0, MgSO4·7H2O, 5.0; sucrose, 1,0; NSG - nutrient solution with 3 salts and glucose (NS3SG) - composition of the nutriente solution (mg/g of support): (NH4)2SO4, 0.943, KH2PO4, 1.0, MgSO4·7H2O, 5.0; glucose, 1,0; DW - hydration solution (distilled water); NS - nutrient solution with 7 salts (NS7S) - Composition of the nutrient solution (mg/g of support): (NH4)2SO4, 0.943; yeast extract 1.0; MgSO4·7H2O, 5; KH2PO4, 1.0; KCl, 0.5; CaCl2·2H2O, 0.008; FeSO4·7H2O, 0.01; ZnSO4·7H2O, 0.001; NSS - nutrient solution with 7 salts and sucrose (NS7SS) - Composition of the nutrient solution (mg/g of support): (NH4)2SO4, 0.943; yeast extract 1.0; MgSO4·7H2O, 5; KH2PO4, 1.0; KCl, 0.5; CaCl2·2H2O, 0.008; FeSO4·7H2O, 0.01; ZnSO4·7H2O, 0.001; sucrose, 1,0. NSG - nutrient solution with 7 salts and glucose (NS7SG) Composition of the nutrient solution (mg/g of support): (NH4)2SO4, 0.943; yeast extract 1.0; MgSO4·7H2O, 5; KH2PO4, 1.0; KCl, 0.5; CaCl2·2H2O, 0.008; FeSO4·7H2O, 0.01; ZnSO4·7H2O, 0.001; glucose, 1,0.

S2.4. Laccase activity of *Geotrichum candidum* using ABTS method and using guaiacol

Table S4 - Enzymatic activity in babassu mesocarp fermented with *G. candidum*, using ABTS and guaiacol as substrate.

Laccases Activity (U.mL^{-1})		
Type of	substrate	Hydration Solution

support		3 salts			DW	7 salts		
		NS	NSS	NSG		NS	NSS	NSG
**IN-BM**	ABTS	0.086± 0.006	0.308± 0.040	0.336± 0.037	0.039± 0.003	0.104± 0.052	0.347± 0.049	0.361± 0.048
	Guaiacol	0.053± 0.004	0.198± 0.027	0.221± 0.028	0.028± 0.004	0.072± 0.043	0.223± 0.046	0.249± 0.035
**DEF-BM**	ABTS	0.117± 0.008	0.389± 0.027	0.408± 0.044	0.062± 0.002	0.134± 0.039	0.419± 0.030	0.441± 0.044
	Guaiacol	0.084± 0.006	0.277± 0.007	0.302± 0.002	0.048± 0.003	0.103± 0.008	0.298± 0.002	0.326± 0.005

Caption: IN-BM (in natura babassu mesocarp); DEF-BM (defatted babassu mesocarp); NS - (nutrient solution with 3 salts (NS3S) – composition of the nutriente solution (mg/g of support): $(NH4)_2SO_4$, 0.943, KH_2PO_4, 1.0, $MgSO_4·7H_2O$, 5.0; NSS - nutrient solution with 3 salts and sucrose (NS3SS) - composition of the nutriente solution (mg/g of support): $(NH4)_2SO_4$, 0.943, KH_2PO_4, 1.0, $MgSO_4·7H_2O$, 5.0; sucrose, 1,0; NSG - nutrient solution with 3 salts and glucose (NS3SG) - composition of the nutriente solution (mg/g of support): $(NH4)_2SO_4$, 0.943, KH_2PO_4, 1.0, $MgSO_4·7H_2O$, 5.0; glucose, 1,0; DW - hydration solution (distilled water); NS - nutrient solution with 7 salts (NS7S) - Composition of the nutrient solution (mg/g of support): $(NH4)_2SO_4$, 0.943; yeast extract 1.0; $MgSO_4·7H_2O$, 5; KH_2PO_4, 1.0; KCl, 0.5; $CaCl_2·2H_2O$, 0.008; $FeSO_4·7H_2O$, 0.01; $ZnSO_4·7H_2O$, 0.001; NSS - nutrient solution with 7 salts and sucrose (NS7SS) - Composition of the nutrient solution (mg/g of support): $(NH4)_2SO_4$, 0.943; yeast extract 1.0; $MgSO_4·7H2O$, 5; KH_2PO_4, 1.0; KCl, 0.5; $CaCl_2·2H_2O$, 0.008; $FeSO_4·7H_2O$, 0.01; $ZnSO_4·7H_2O$, 0.001; sucrose, 1,0. NSG - nutrient solution with 7 salts and glucose (NS7SG) Composition of the nutrient solution (mg/g of support): $(NH4)_2SO_4$, 0.943; yeast extract 1.0; $MgSO_4·7H_2O$, 5; KH_2PO_4, 1.0; KCl, 0.5; $CaCl_2·2H_2O$, 0.008; $FeSO_4·7H_2O$, 0.01; $ZnSO_4·7H_2O$, 0.001; glucose, 1,0.

S2.5. Enzyme production kinetics

The production kinetics of lipases and lacases produced, respectively, by *G. candidum* and *T. harzianum* in babassu mesocarp were evaluated for 192 hours in order to identify the optimum production time for the enzymes under the conditions used in this study (table S5).

Table S5 - Kinetics of lipase and laccase production with babassu mesocarp supplemented with SN7SG.

Table of lipase and laccase production kinetics in babassu mesocarp			
Lipase – *G. candidum*		Laccase – *T. harzianum*	
Time (h)	Activity ($U.mL^{-1}$)	Time (h)	Activity ($U.mL^{-1}$)
0	0	0	0
24	4.63±0.09	24	0.126±0.018
48	12.72±0.13	48	0.224±0.037
72	20.94±1.36	72	0.408±0.064
96	28.86±1.54	96	0.541±0.064
120	35.54±0.87	120	0.612±0.044

144	39.92±2.04	144	0,702±0.089
168	40.11±0.61	168	0.719±0.097
192	40.74±1.27	192	0.691±0.021

Caption: Lipase and laccase activity on different days of cultivation monitored over 192 hours. Cultivation conditions for lipase production: in natura babassu mesocarp (IN-BM) moistened with SN7SG (nutrient solution with 7 salts and glucose) - Composition of the nutrient solution (mg/g of support): $(NH4)_2SO_4$, 0.943; yeast extract 1.0; $MgSO_4 \cdot 7H_2O$, 5; KH_2PO_4, 1.0; KCl, 0.5; $CaCl_2 \cdot 2H_2O$, 0.008; $FeSO_4 \cdot 7H_2O$, 0.01; $ZnSO_4 \cdot 7H_2O$, 0.001; glucose, 1,0; temperature 30°C; humidity 90%. Analysis method: titulometric method. Cultivation conditions for laccase production: defatted babassu mesocarp (DEF-BM) moistened SN7SG (nutrient solution with 7 salts and glucose) - Composition of the nutrient solution (mg/g of support): $(NH4)_2SO_4$, 0.943; yeast extract 1.0; $MgSO_4 \cdot 7H_2O$, 5; KH_2PO_4, 1.0; KCl, 0.5; $CaCl_2 \cdot 2H_2O$, 0.008; $FeSO_4 \cdot 7H_2O$, 0.01; $ZnSO_4 \cdot 7H_2O$, 0.001; glucose, 1,0; temperature 30°C; humidity 90%. Analysis method: ABTS method.

S3. Statistical Test

Student's t-test (Table S6) was used, using the analysis of two paired means to statistically analyze the results. A comparison was made between the best results using IN-BM (in natura babassu mesocarp) and DEF-BM (defatted babassu mesocarp) with NS7SG for the production of the lipase enzyme and a comparison between IN-BM and DEF-BM with NS7SG for the production of the laccase enzyme. The same test was then carried out comparing the best results for the production of each of the enzymes (lipase and laccase) in IN-BM using NS3SG, NS3SS, NS7SG and NS7SS and in DEF-BM also using NS3SG, NS3SS, NS7SG and NS7SS.

Table S6. Statistical analysis of nutrition methods, identification and quantification of lipase and laccase

Analysis of Lipase and Laccase identification methods

IN-BM - NS7SG - Lipase

	Titulometric	*p*-nitro phenol
1	32,91	29,07
2	33,10	28,89
3	33,41	28,68
Mean	33,14	28,88
Median	33,1	28,89

F-test: two samples for variances

	Titulometric	*p*-nitro phenol
Mean	33,14	28,88
Variance	0,0637	0,0381
Observations	3	3
gl	2	2
F	1,67191601	
P(F<=f) uni-caudal	0,374263261	
F critical uni-caudal	19	

F-test
H0 = Equal variance
H1 = Different variances

T-test: two paired samples for averages

	Titulomeric	*p*-nitro phenol
Mean	33,14	28,88
Variance	0,0637	0,0381
Observations	3	3
Correlação de Pearson	-0,99565025	
Hipótese da diferença de média	0	
gl	2	
Stat t	16,50303533	
P(T<=t) uni-caudal	0,001825822	
t critical uni-caudal	2,91998558	
P(T<=t) bi-caudal	0,003651644	
t critical bi-caudal	4,30265273	

Paired T-test
H0 = Equal means
H1 = Different means

DEF-BM - NS7SG - Lipase

	Titulometric	*p*-nitro phenol
1	26,98	22,81
2	26,74	22,93
3	27,97	23,11
Mean	27,23	22,95
Median	26,98	22,93

F-test: two samples for variances

	Titulometric	*p*-nitro phenol
Mean	27,23	22,95
Variance	0,4251	0,0228
Observations	3	3
gl	2	2
F	18,64473684	
P(F<=f) uni-caudal	0,05090422	
F critical uni-caudal	19	

T-test: two paired samples for averages

	Titulomeric	*p*-nitro phenol
Mean	27,23	22,95
Variance	0,4251	0,0228
Observations	3	3
Correlação de Pearson	0,828852368	
Hipótese da diferença de média	0	
gl	2	
Stat t	13,89346793	
P(T<=t) uni-caudal	0,002570335	
t critical uni-caudal	2,91998558	
P(T<=t) bi-caudal	0,00514067	
t critical bi-caudal	4,30265273	

F-test
H0 = Equal variance
H1 = Different variances

Paired T-test
H0 = Equal means
H1 = Different means

IN-BM - NS7SG - Laccase

	ABTS	Guaiacol
1	0,439	0,358
2	0,412	0,347
3	0,502	0,333
Mean	0,451	0,346
Median	0,439	0,347

F-test: two samples for variances

	ABTS	*Guaiacol*
Mean	0,451	0,346
Variance	0,0000370	0,000157
Observations	3	3
gl	2	2
F	0,23566879	
P(F<=f) uni-caudal	0,190721649	
F critical uni-caudal	0,052631579	

T-test: two paired samples for averages

	ABTS	*Guaiacol*
Mean	0,451	0,346
Variance	0,0000370	0,000157
Observations	3	3
Correlação de Pearson	0,150885377	
Hipótese da diferença de média	0	
gl	2	
Stat t	13,90758975	
P(T<=t) uni-caudal	0,002565158	
t critical uni-caudal	2,91998558	
P(T<=t) bi-caudal	0,005130316	
t critical bi-caudal	4,30265273	

F-test
H0 = Equal variance
H1 = Different variances

Paired T-test
H0 = Equal means
H1 = Different means

DEF-BM - NS7SG - Laccase

	ABTS	Guaiacol
1	0,535	0,411
2	0,539	0,413
3	0,498	0,415
Mean	0,524	0,413
Median	0,535	0,413

F-test: two samples for variances

	ABTS	*Guaiacol*
Mean	0,524	0,413
Variance	0,000144	0,0000040
Observations	3	3
gl	2	2
F	36	
P(F<=f) uni-caudal	0,027027027	
F critical uni-caudal	19	

T-test: two paired samples for averages

	ABTS	*Guaiacol*
Mean	0,524	0,413
Variance	0,000144	0,0000040
Observations	3	3
Correlação de Pearson	-0,5	
Hipótese da diferença de média	0	
gl	2	
Stat t	14,65950759	
P(T<=t) uni-caudal	0,002310536	
t critical uni-caudal	2,91998558	
P(T<=t) bi-caudal	0,004621072	
t critical bi-caudal	4,30265273	

F-test
H0 = Equal variance
H1 = Different variances

Paired T-test
H0 = Equal means
H1 = Different means

Geotrichum candidum - Lipase

IN-BM - Titulometric method

	SN7SG	SN7SS
1	32,91	29,72
2	33,10	30,17
3	33,41	30,92
Mean	33,14	30,27
Median	33,1	30,17

F-test: two samples for variances

	SN7SG	*SN7SS*
Mean	33,14	30,27
Variance	0,0637	0,3675
Observations	3	3
gl	2	2
F	0,173333333	
P(F<=f) uni-caudal	0,147727273	
F critical uni-caudal	0,052631579	

T-test: two paired samples for averages

	SN7SG	*SN7SS*
Mean	33,14	30,27
Variance	0,0637	0,3675
Observations	3	3
Correlação de Pearson	0,999983981	
Hipótese da diferença de média	0	
gl	2	
Stat t	14,04883655	
P(T<=t) uni-caudal	0,002514224	
t critical uni-caudal	2,91998558	
P(T<=t) bi-caudal	0,005028447	
t critical bi-caudal	4,30265273	

F-test
H0 = Equal variance
H1 = Different variances

Paired T-test
H0 = Equal means
H1 = Different means

IN-BM - Titulometric method

	NS3SG	NS3SS
1	28,98	26,76
2	29,82	26,08
3	30,72	28,04
Mean	29,84	26,96
Median	29,82	26,76

F-test: two samples for variances

	NS3SG	NS3SS
Mean	29,84	26,96
Variance	0,7572	0,9904
Observations	3	3
gl	2	2
F	0,76453958	
P(F<=f) uni-caudal	0,433279927	
F critical uni-caudal	0,052631579	

T-test: two paired samples for averages

	NS3SG	NS3SS
Mean	29,84	26,96
Variance	0,7572	0,9904
Observations	3	3
Correlação de Pearson	0,658209638	
Hipótese da diferença de média	0	
gl	2	
Stat t	6,399473316	
P(T<=t) uni-caudal	0,011779313	
t critical uni-caudal	2,91998558	
P(T<=t) bi-caudal	0,023558625	
t critical bi-caudal	4,30265273	

F-test
H0 = Equal variance
H1 = Different variances

Paired T-test
H0 = Equal means
H1 = Different means

DEF-BM - Titulometric method

	NS7SG	NS7SS
1	26,98	24,26
2	26,74	23,75
3	27,97	25,01
Mean	27,23	24,34
Median	26,98	24,26

F-test: two samples for variances

	NS7SG	NS7SS
Mean	27,23	24,34
Variance	0,4251	0,4017
Observations	3	3
gl	2	2
F	1,058252427	
P(F<=f) uni-caudal	0,485849057	
F critical uni-caudal	19	

T-test: two paired samples for averages

	NS7SG	NS7SS
Mean	27,23	24,34
Variance	0,4251	0,4017
Observations	3	3
Correlação de Pearson	0,973902461	
Hipótese da diferença de média	0	
gl	2	
Stat t	33,82489154	
P(T<=t) uni-caudal	0,000436444	
t critical uni-caudal	2,91998558	
P(T<=t) bi-caudal	0,000872887	
t critical bi-caudal	4,30265273	

F-test
H0 = Equal variance
H1 = Different variances

Paired T-test
H0 = Equal means
H1 = Different means

DEF-BM - Titulometric method

	NS3SG	NS3SS
1	22,83	18,74
2	21,50	19,67
3	24,34	21,35
Mean	22,89	19,92
Median	22,83	19,67

F-test: two samples for variances

	NS3SG	NS3SS
Mean	22,89	19,92
Variance	2,0191	1,7499
Observations	3	3
gl	2	2
F	1,153837362	
P(F<=f) uni-caudal	0,464287609	
F critical uni-caudal	19	

T-test: two paired samples for averages

	NS3SG	NS3SS
Mean	22,89	19,92
Variance	2,0191	1,7499
Observations	3	3
Correlação de Pearson	0,662823131	
Hipótese da diferença de média	0	
gl	2	
Stat t	4,551846643	
P(T<=t) uni-caudal	0,022514621	
t critical uni-caudal	2,91998558	
P(T<=t) bi-caudal	0,045029242	
t critical bi-caudal	4,30265273	

F-test
H0 = Equal variance
H1 = Different variances

Paired T-test
H0 = Equal means
H1 = Different means

Geotrichum candidum - Laccase

IN-BM - ABTS method

	NS7SG	NS7SS
1	0,339	0,311
2	0,328	0,327
3	0,416	0,403
Mean	0,361	0,347
Median	0,339	0,327

F-test: two samples for variances

	NS7SG	NS7SS
Mean	0,361	0,347
Variance	0,002299	0,002416
Observations	3	3
gl	2	2
F	0,951572848	
P(F<=f) uni-caudal	0,487592789	
F critical uni-caudal	0,052631579	

T-test: two paired samples for averages

	NS7SG	NS7SS
Mean	0,361	0,347
Variance	0,002299	0,002416
Observations	3	3
Correlação de Pearson	0,961483764	
Hipótese da diferença de média	0	
gl	2	
Stat t	1,792516319	
P(T<=t) uni-caudal	0,107459492	
t critical uni-caudal	2,91998558	
P(T<=t) bi-caudal	0,214918984	
t critical bi-caudal	4,30265273	

F-test
H0 = Equal variance
H1 = Different variances

Paired T-test
H0 = Equal means
H1 = Different means

IN-BM - ABTS method

	NS3SG	NS3SS
1	0,339	0,274
2	0,298	0,298
3	0,371	0,352
Mean	0,336	0,308
Median	0,339	0,298

F-test: two samples for variances

	NS3SG	*NS3SS*
Mean	0,336	0,308
Variance	0,001339	0,001596
Observations	3	3
gl	2	2
F	0,838972431	
P(F<=f) uni-caudal	0,456218058	
F critical uni-caudal	0,052631579	

T-test: two paired samples for averages

	NS3SG	*NS3SS*
Mean	0,336	0,308
Variance	0,001339	0,001596
Observations	3	3
Correlação de Pearson	0,621809211	
Hipótese da diferença de média	0	
gl	2	
Stat t	1,451082392	
P(T<=t) uni-caudal	0,141926948	
t critical uni-caudal	2,91998558	
P(T<=t) bi-caudal	0,283853896	
t critical bi-caudal	4,30265273	

F-test
H0 = Equal variance
H1 = Different variances

Paired T-test
H0 = Equal means
H1 = Different means

DEF-BM - ABTS method

	NS7SG	NS7SS
1	0,397	0,389
2	0,442	0,419
3	0,484	0,449
Mean	0,441	0,419
Median	0,442	0,419

F-test: two samples for variances

	NS7SG	*NS7SS*
Mean	0,441	0,419
Variance	0,001893	0,0009
Observations	3	3
gl	2	2
F	2,103333333	
P(F<=f) uni-caudal	0,322234157	
F critical uni-caudal	19	

T-test: two paired samples for averages

	NS7SG	*NS7SS*
Mean	0,441	0,419
Variance	0,001893	0,0009
Observations	3	3
Correlação de Pearson	0,999801882	
Hipótese da diferença de média	0	
gl	2	
Stat t	2,816811359	
P(T<=t) uni-caudal	0,053155545	
t critical uni-caudal	2,91998558	
P(T<=t) bi-caudal	0,10631109	
t critical bi-caudal	4,30265273	

F-test
H0 = Equal variance
H1 = Different variances

Paired T-test
H0 = Equal means
H1 = Different means

DEF-BM - ABTS method

	NS3SG	NS3SS
1	0,371	0,59
2	0,397	0,397
3	0,456	0,411
Mean	0,408	0,466
Median	0,397	0,411

F-test: two samples for variances

	NS3SG	*NS3SS*
Mean	0,408	0,466
Variance	0,001897	0,011581
Observations	3	3
gl	2	2
F	0,16380278	
P(F<=f) uni-caudal	0,140747885	
F critical uni-caudal	0,052631579	

T-test: two paired samples for averages

	NS3SG	*NS3SS*
Mean	0,408	0,466
Variance	0,001897	0,011581
Observations	3	3
Correlação de Pearson	-0,69008162	
Hipótese da diferença de média	0	
gl	2	
Stat t	-0,71129512	
P(T<=t) uni-caudal	0,275335511	
t critical uni-caudal	2,91998558	
P(T<=t) bi-caudal	0,550671021	
t critical bi-caudal	4,30265273	

F-test
H0 = Equal variance
H1 = Different variances

Paired T-test
H0 = Equal means
H1 = Different means

Trichoderma harzianum - Lipase

IN-BM - Titulometric method

	NS7SG	NS7SS
1	25,85	22,32
2	26,47	22,93
3	26,09	24,08
Mean	26,14	23,11
Median	26,09	22,93

F-test: two samples for variances

	NS7SG	*NS7SS*
Mean	26,14	23,11
Variance	0,097733333	0,7987
Observations	3	3
gl	2	2
F	0,122365511	
P(F<=f) uni-caudal	0,109024653	
F critical uni-caudal	0,052631579	

T-test: two paired samples for averages

	NS7SG	*NS7SS*
Mean	26,14	23,11
Variance	0,097733333	0,7987
Observations	3	3
Correlação de Pearson	0,216899937	
Hipótese da diferença de média	0	
gl	2	
Stat t	5,954002355	
P(T<=t) uni-caudal	0,01353426	
t critical uni-caudal	2,91998558	
P(T<=t) bi-caudal	0,027068519	
t critical bi-caudal	4,30265273	

F-test
H0 = Equal variance
H1 = Different variances

Paired T-test
H0 = Equal means
H1 = Different means

IN-BM - Titulometric method

	NS3SG	NS3SS
1	22,49	19,72
2	23,51	20,07
3	24,86	21,56
Mean	23,62	20,45
Median	23,51	20,07

F-test: two samples for variances

	NS3SG	NS3SS
Mean	23,62	20,45
Variance	1,4133	0,9547
Observations	3	3
gl	2	2
F	1,480360323	
P(F<=f) uni-caudal	0,40316723	
F critical uni-caudal	19	

T-test: two paired samples for averages

	NS3SG	NS3SS
Mean	23,62	20,45
Variance	1,4133	0,9547
Observations	3	3
Correlação de Pearson	0,96553495	
Hipótese da diferença de média	0	
gl	2	
Stat t	15,5359806	
P(T<=t) uni-caudal	0,002058751	
t critical uni-caudal	2,91998558	
P(T<=t) bi-caudal	0,004117503	
t critical bi-caudal	4,30265273	

F-test
H0 = Equal variance
H1 = Different variances

Paired T-test
H0 = Equal means
H1 = Different means

DEF-BM - Titulometric method

	NS7SG	NS7SS
1	18,25	16,49
2	19,72	17,42
3	22,11	18,65
Mean	20,03	17,52
Median	19,72	17,42

F-test: two samples for variances

	NS7SG	NS7SS
Mean	20,03	17,52
Variance	3,795433333	1,1739
Observations	3	3
gl	2	2
F	3,233182838	
P(F<=f) uni-caudal	0,23622887	
F critical uni-caudal	19	

T-test: two paired samples for averages

	NS7SG	NS7SS
Mean	20,03	17,52
Variance	3,795433333	1,1739
Observations	3	3
Correlação de Pearson	0,998391191	
Hipótese da diferença de média	0	
gl	2	
Stat t	4,998250268	
P(T<=t) uni-caudal	0,018887253	
t critical uni-caudal	2,91998558	
P(T<=t) bi-caudal	0,037774507	
t critical bi-caudal	4,30265273	

F-test
H0 = Equal variance
H1 = Different variances

Paired T-test
H0 = Equal means
H1 = Different means

DEF-BM - Titulometric method

	NS3SG	NS3SS
1	17,21	15,93
2	18,88	14,78
3	19,95	16,96
Mean	18,68	15,89
Median	18,88	15,93

F-test: two samples for variances

	NS3SG	NS3SS
Mean	18,68	15,89
Variance	1,9069	1,1893
Observations	3	3
gl	2	2
F	1,60338014	
P(F<=f) uni-caudal	0,384116013	
F critical uni-caudal	19	

T-test: two paired samples for averages

	NS3SG	NS3SS
Mean	18,68	15,89
Variance	1,9069	1,1893
Observations	3	3
Correlação de Pearson	0,35794754	
Hipótese da diferença de média	0	
gl	2	
Stat t	3,401680257	
P(T<=t) uni-caudal	0,038309742	
t critical uni-caudal	2,91998558	
P(T<=t) bi-caudal	0,076619483	
t critical bi-caudal	4,30265273	

F-test
H0 = Equal variance
H1 = Different variances

Paired T-test
H0 = Equal means
H1 = Different means

Trichoderma harzianum - Laccase

IN-BM - ABTS method

	NS7SG	NS7SS
1	0,439	0,387
2	0,412	0,437
3	0,502	0,502
Mean	0,451	0,442
Median	0,439	0,44

F-test: two samples for variances

	NS7SG	NS7SS
Mean	0,451	0,442
Variance	0,002133	0,003325
Observations	3	3
gl	2	2
F	0,641503759	
P(F<=f) uni-caudal	0,390802492	
F critical uni-caudal	0,052631579	

T-test: two paired samples for averages

	NS7SG	NS7SS
Mean	0,451	0,442
Variance	0,002133	0,003325
Observations	3	3
Correlação de Pearson	0,735039229	
Hipótese da diferença de média	0	
gl	2	
Stat t	0,396844325	
P(T<=t) uni-caudal	0,364912164	
t critical uni-caudal	2,91998558	
P(T<=t) bi-caudal	0,729824329	
t critical bi-caudal	4,30265273	

F-test
H0 = Equal variance
H1 = Different variances

Paired T-test
H0 = Equal means
H1 = Different means

IN-BM - ABTS method

	NS3SG	NS3SS
1	0,377	0,365
2	0,429	0,408
3	0,502	0,514
Mean	0,436	0,429
Median	0,429	0,408

F-test: two samples for variances

	NS3SG	*NS3SS*
Mean	0,436	0,429
Variance	0,003943	0,005881
Observations	3	3
gl	2	2
F	0,670464207	
P(F<=f) uni-caudal	0,401364007	
F critical uni-caudal	0,052631579	

T-test: two paired samples for averages

	NS3SG	*NS3SS*
Mean	0,436	0,429
Variance	0,003943	0,005881
Observations	3	3
Correlação de Pearson	0,989830015	
Hipótese da diferença de média	0	
gl	2	
Stat t	0,710742316	
P(T<=t) uni-caudal	0,275474895	
t critical uni-caudal	2,91998558	
P(T<=t) bi-caudal	0,550949791	
t critical bi-caudal	4,30265273	

F-test
H0 = Equal variance
H1 = Different variances

Paired T-test
H0 = Equal means
H1 = Different means

DEF-BM - ABTS method

	NS7SG	NS7SS
1	0,425	0,435
2	0,534	0,522
3	0,613	0,576
Mean	0,524	0,511
Median	0,534	0,522

F-test: two samples for variances

	NS7SG	*NS7SS*
Mean	0,524	0,511
Variance	0,008911	0,005061
Observations	3	3
gl	2	2
F	1,760719225	
P(F<=f) uni-caudal	0,362224449	
F critical uni-caudal	19	

T-test: two paired samples for averages

	NS7SG	*NS7SS*
Mean	0,524	0,511
Variance	0,008911	0,005061
Observations	3	3
Correlação de Pearson	0,999099558	
Hipótese da diferença de média	0	
gl	2	
Stat t	0,957505801	
P(T<=t) uni-caudal	0,219678247	
t critical uni-caudal	2,91998558	
P(T<=t) bi-caudal	0,439356494	
t critical bi-caudal	4,30265273	

F-test
H0 = Equal variance
H1 = Different variances

Paired T-test
H0 = Equal means
H1 = Different means

DEF-BM - ABTS method

	NS3SG	NS3SS
1	0,434	0,415
2	0,492	0,457
3	0,586	0,574
Mean	0,504	0,482
Median	0,492	0,457

F-test: two samples for variances

	NS3SG	*NS3SS*
Mean	0,504	0,482
Variance	0,005884	0,006789
Observations	3	3
gl	2	2
F	0,866696126	
P(F<=f) uni-caudal	0,464294169	
F critical uni-caudal	0,052631579	

T-test: two paired samples for averages

	NS3SG	*NS3SS*
Mean	0,504	0,482
Variance	0,005884	0,006789
Observations	3	3
Correlação de Pearson	0,991563332	
Hipótese da diferença de média	0	
gl	2	
Stat t	3,232033905	
P(T<=t) uni-caudal	0,041931676	
t critical uni-caudal	2,91998558	
P(T<=t) bi-caudal	0,083863352	
t critical bi-caudal	4,30265273	

F-test
H0 = Equal variance
H1 = Different variances

Paired T-test
H0 = Equal means
H1 = Different means
